# Alcohol-induced bone loss driven by dysregulated spatial distribution of gut microbiota and PGD2-IL17 pathway-mediated osteoclast activation

**DOI:** 10.3389/fmicb.2025.1551028

**Published:** 2025-05-15

**Authors:** Haoyu Guo, Yujing Bi, Gongzi Zhang, Shicheng Luo, Xiaopeng Jia, Ruifu Yang, Ye Peng, Lihai Zhang

**Affiliations:** ^1^School of Medicine, Nankai University, Tianjin, China; ^2^Department of Orthopedics, Chinese PLA General Hospital, Beijing, China; ^3^State Key Laboratory of Pathogen and Biosecurity, Academy of Military Medical Sciences, Beijing, China; ^4^Department of Rehabilitation, Chinese PLA General Hospital, Beijing, China; ^5^School of Medicine, Jinzhou Medical University, Jinzhou, China

**Keywords:** alcohol, bone loss, microbiota, segmental gut, precision medicine, prostaglandin D2, Th17 cells

## Abstract

**Introduction:**

Alcohol-induced damage to bone microstructure leads to alcoholic osteoporosis (AOP). While prior studies have demonstrated alcohol’s negative impact on bone density, the mechanisms by which alcohol induces osteoporosis through immune pathways, gut microbiota dysbiosis, and metabolic alterations remain insufficiently characterized. Given that alcohol is primarily absorbed in the upper gastrointestinal tract, in this research, we aimed to elucidate the role of spatial distribution disorders in gut microbiota and metabolites in the pathogenesis of alcohol-induced osteoporosis. We further sought to evaluate the potential of microbiota supplementation and targeted immunosuppressants as therapeutic strategies for related bone diseases.

**Methods:**

An osteoporosis model using mice was established using alcohol drinking bottles, and bone loss was validated using micro-computed tomography. Segmented intestinal samples and fecal samples were analyzed using 16S rRNA sequencing and metabolomics. Mechanistic studies were conducted by supplementing *R. intestinalis*, prostaglandin D2 (PGD2), and its specific immune inhibitor, ramatroban. Analytical methods included tartrate-resistant acid phosphatase staining, flow cytometry, and enzyme-linked immunosorbent assay.

**Results:**

Alcohol disrupted the spatial complexity of intestinal segments and fecal microbiota in mice, causing metabolic dysregulation and ultimately leading to elevated PGD2 levels. This, in turn, triggered Th17/Treg immune imbalance and osteoclast activation, resulting in bone loss. Supplementation with the probiotic *R. intestinalis* or inhibition of PGD2 significantly improved bone density and alleviate inflammation.

**Conclusion:**

This study demonstrates that alcohol-induced elevation of PGD2 is a key pathogenic factor in AOP. PGD2 accelerates bone loss by promoting osteoclast formation through the activation of Th17 cells. Furthermore, this study highlights the importance of investigating the spatial distribution of gut microbiota and metabolites, providing potential targets and novel strategies for the precise treatment of AOP and other diseases associated with external stimuli.

## Graphical abstract

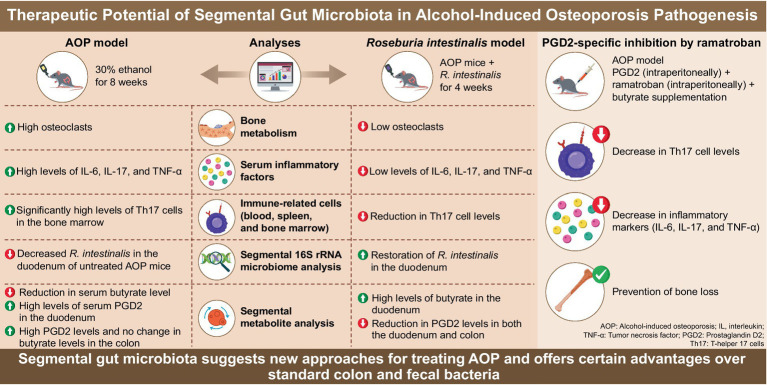

## 1 Introduction

The gut microbiota comprises a community of microorganisms and their surrounding environment, and its stability is crucial for maintaining health (Cheng et al., 2022). Current research on microbiota and metabolites predominantly focuses on the lower gastrointestinal (GI) tract, such as the colon or feces, where microbial abundance is higher (Nardone et al., 2017). In contrast, the upper GI tract, characterized by unique anatomical features and lower microbial abundance, has received comparatively little attention (She et al., 2024). As the transit station for nutrient digestion and absorption in the human body, the significance of research on gut microbiota under the perturbations of external factors and diet has been well established (Zmora et al., 2019; Bourdeau-Julien et al., 2023). However, currently, there is relatively little research attention on how the microbiota and metabolites in different intestinal segments are affected by external factors during the occurrence and development of diseases and whether these changes are related to disease mechanisms (Lawal et al., 2023; Gao et al., 2023). Furthermore, it is still uncertain whether the microbiota and metabolites from the lower GI tract, such as fecal or colonic samples, can reliably represent the entire gut environment (Shalon et al., 2023). This issue has also been increasingly questioned by more and more people with the in-depth research of gut microbiota (Rode et al., 2024). Our research aims to investigate the spatial distribution of the gut microenvironment and its role in alcohol-induced osteoporosis (AOP).

AOP, a prevalent form of secondary osteoporosis, results from prolonged alcohol consumption, leading to bone degradation and increased risk of fracture (Walker-Bone, 2012). Although recent research suggests a gut–bone axis (Zhang et al., 2024) in bone metabolism, the specific connection pathways between alcohol, microbiota, metabolites, and bone loss remains obscure (Cheng et al., 2021). Especially, the spatial distribution of microbiota and metabolites under the influence of alcohol perturbation still requires further exploration (Liu et al., 2022; Zheng et al., 2024).

When studying AOP from the perspective of the gut-bone axis, it is essential to explore the relationship between the upper GI tract and AOP. Firstly, as the area where alcohol first impacts and is absorbed (Sosnowski and Przybyłkowski, 2024), the upper GI tract experiences direct interference from alcohol on its microbiota and metabolites (Wang et al., 2020). Thus, it is necessary and of great significance to identify the characteristic microbiota and metabolites with the highest degree of association with AOP in the upper GI tract. This research can also fill the gap on the relationship between alcohol and the spatial perturbation of the microbiota.

Alcohol can also affect the absorption of nutrients in the upper GI tract. This may ultimately lead to osteoporosis due to nutrient deficiency (Naude et al., 2012). And more importantly, after alcohol disrupts the balance of the microbiota, the dysregulated microbiota and their metabolites, such as certain inflammatory factors or bacterial components, can easily translocate through the damaged intestinal wall. This translocation activates local and even systemic immune responses beneath the intestinal wall or outside the intestine (for instance, immune responses in Peyer’s patches (Hirota et al., 2013), bone marrow, and the spleen), ultimately contributing to the development of AOP.

The immune pathway is the most likely mechanism by which alcohol affects the occurrence and development of AOP through the upper GI tract. As osteoarthritis progresses, inflammatory factors in the patients’ bodies gradually accumulate, and the risk of osteoporosis increases accordingly (Kim et al., 2022). Alcohol intake, largely inflames the intestinal environment and can trigger changes in many inflammatory factors. Therefore, due to inflammation, in AOP, the treatment regimens for osteoarthritis patients can be significantly referenced. For instance, non-steroidal anti-inflammatory drugs (NSAIDs) or targeted drugs can be used to inhibit inflammatory factors and non-oral treatment methods like Gutong Patch can be adopted to prevent AOP (Wang et al., 2023). Also, drugs that can regulate the metabolism of intestinal flora or affect the intestinal microenvironment can be used to inhibit the progression of AOP (Zhu et al., 2022). Moreover, based on the research findings, treatment regimens targeting gut microbiota, metabolites, or environment of the upper GI tract can be proposed or developed. This will enable the implementation of precise treatment for AOP in a more meticulous manner.

The pathogenesis of AOP may be associated with inflammatory immune mechanisms involving tumor necrosis factor (TNF)-*α*, interleukin (IL)-1β, IL-6, IL-10, and IL-17 (Lowe et al., 2018). These factors may exert their effects through interactions with various bone-related cells. Regarding IL-1β, it can trigger the activation of the natural killer-κB pathway, leading to the downregulation of miR-506 expression. In osteosarcoma, this process promotes cell growth through JAG1 (Hu et al., 2017). Therefore, in AOP, a similar molecular cascade is highly likely to disrupt the normal functions of osteoblasts and osteoclasts by interfering with the gene regulation involved in bone remodeling.

In addition to regulating osteoblasts and osteoclasts that maintain bone balance, directly regulating the activity of bone marrow mesenchymal stem cells and thus affecting osteoblast-induced bone formation, may also contribute to the occurrence of AOP. Research findings based on the osteoporosis in female mice show that the miR-665/SOST axis plays a crucial role in regulating the phenotypes of bone marrow mesenchymal stem cells and the development of osteoporosis symptoms (Zeng et al., 2024). Currently, whether alcohol consumption can interfere with the miR-665/SOST axis, resulting in abnormal differentiation of bone marrow mesenchymal stem cells and affecting bone homeostasis, remains unclear. However, it can be speculated that such abnormalities are likely to change the balance between bone formation and bone resorption, promoting the development of AOP.

Furthermore, previous studies have found that in inflammatory bowel disease (IBD), which is closely related to alcohol intake, Th17 TNF-*α*(+) cells in the PP lymph nodes are significantly activated and migrate to the bone marrow, inducing the differentiation of osteoclasts and ultimately leading to bone destruction (Ciucci et al., 2015). Considering the above-mentioned research, we can explore how alcohol-induced inflammation and subsequent molecular changes trigger AOP. We aimed to elucidate the role of spatial distribution disorders in gut microbiota and metabolites in the pathogenesis of AOP.

## 2 Materials and methods

### 2.1 *In vivo* mouse model

#### 2.1.1 AOP model induction

Male C57BL/6 mice aged 8 weeks were obtained from SiPeiFu Biotechnology Co., Ltd. (Beijing, China). The study was approved by the Institutional Animal Care and Use Committee (IACUC) of Beijing Yaokang Biotechnology Co., Ltd. (Protocol Number: GPT-BJAP002). All experimental procedures adhered strictly to the “3R” principles and were conducted under IACUC supervision, ensuring humane care for all animals in compliance with ARRIVE guidelines. The mice were housed under standard conditions (22 ± 2°C, 50 ± 15% humidity, 12-h light/dark cycle). Alcohol was administered via alcohol drinking bottles at concentrations of 15 and 30% for 8 consecutive weeks (Yang et al., 2014). The groups are subsequently referred to as the 15%-AOP group and the 30%-AOP group. The alcohol solution was refreshed every 2 days to ensure consistent concentration. PGD2, ramatroban (Pang et al., 2021), and butyrate were used as previously described during the AOP modeling process (Chen et al., 2020). The gavage concentrations were 0.12 mg/mL for PGD2 and ramatroban, and 0.05 g/mL for butyrate (Tyagi et al., 2018), with approximately 0.1 mL administered per gavage. Food intake was recorded and converted to energy intake [alcohol, 7 kcal/g (Traversy and Chaput, 2015); feed, 5.24 kcal/g], and the energy-to-weight conversion rate (total energy intake/total weight gain) was calculated.

#### 2.1.2 Gut segment sampling

Mice were anesthetized with 1% pentobarbital (50 mg/kg) before sampling. Three-centimeter segments of the duodenum, jejunum, ileum, and colon were collected. The metabolites were scraped off, and the intestinal segments were rinsed clean. The metabolites, remaining intestinal segments, and feces were stored in 1.5-mLsterile cryotubes.

#### 2.1.3 *Roseburia intestinalis* supplementation experiment

*Roseburia intestinalis* (JCM Catalogue, Tsukuba, Ibaraki, Japan) was anaerobically cultured at 37°C. After 8 weeks of alcohol administration in mice, alcohol was discontinued, and *R. intestinalis* was administered via gavage for 4 weeks. Each supplementation with *R. intestinalis* was at a concentration of 2 × 10^8^ CFU. At the end of the experiment (week 12), samples were collected for comparison before and after microbiota treatment (Seo et al., 2020).

### 2.2 Tartrate-resistant acid phosphatase (TRAP) staining

Femurs were decalcified in 15% ethylenediaminetetraacetic acid, embedded in paraffin, sectioned, and stained to assess for TRAP activity (Song et al., 2019). Osteoclasts were counted in 20× magnification fields using Image-Pro Plus 6.0 software (Media Cybernetics, Inc., Rockville, MD, United States).

### 2.3 Micro-computed tomography (micro-CT) analyses

After dissection, femurs were fixed in 4% paraformaldehyde and scanned using micro-CT. The tibial analysis focused on the trabecular bone, defined as the region extending from the proximal end (1% of the total bone length) to the growth plate (Liu et al., 2019). The trabecular bone mineral density (BMD), bone volume/tissue volume (BV/TV), bone surface/bone volume (BS/BV), trabecular number (Tb.N), trabecular separation (Tb.Sp), and trabecular thickness (Tb.Th) were analyzed.

### 2.4 Flow cytometry analysis

Single-cell suspensions from blood, spleen, and bone marrow were prepared through enzymatic digestion. Flow cytometry was employed to analyze the proportions of CD3(+)CD4(+) T cells, TNF-*α*(+)CD4(+) T cells, IL-17(+)CD4(+) T cells (Komatsu et al., 2014), and Foxp3(+)CD25A(+) T cells (Yuan et al., 2017).

### 2.5 Biochemical analysis of serum parameters

Serum was collected by centrifugation, and the levels of osteocalcin (OC), alkaline phosphatase (ALP), procollagen type I N-terminal pro-peptide (P1NP), TRAP-5b, receptor activator of nuclear factor kappa beta ligand (RANKL), C-terminal telopeptide of type I collagen (CTX-1) (Elghareeb et al., 2022), IL-6, TNF-α, IL-17, albumin (ALB) (Gioulbasanis et al., 2011), lipopolysaccharide (LPS) (Stephens and von der Weid, 2020), and diamine oxidase (DAO) (Zhang et al., 2014) were measured using specific kits following the manufacturer’s instructions. All kits were obtained from Abbkine Biotechnology Co., Ltd. (Wuhan, Hubei, China).

### 2.6 16S rRNA microbiome sequencing

*Processing intestinal and fecal samples*: After sacrificing the mice, use sterilized surgical instruments to open the abdominal cavity and expose the intestines. Carefully separate the entire intestinal segment from the start of the duodenum to the lower section of the cecum with sterile ophthalmic scissors and place it on sterile gauze. Cut approximately 2–3 cm long intestinal segments from the middle of the duodenum, middle of the jejunum, middle of the ileum, and colon segment, respectively. Collect 3–4 fresh feces of mice using a mouse feces collection box or from the lower end of the rectum.

*Samples for 16S rRNA sequencing*: Rinse the intestinal segments thoroughly with phosphate buffer saline. Put the intestinal segments and fresh feces into 1.5 mL sterile cryotubes separately, quickly freeze them in liquid nitrogen, and store them in a −80°C refrigerator. Place the samples according to the experimental groups and numbers for easy retrieval and use in the subsequent steps.

The cryotubes were taken out from the −80°C freezer, and the samples were thawed on ice. Each intestinal segment (duodenum, jejunum, ileum, and colon) and fecal sample was placed in a sterile mortar, and liquid nitrogen was added before grinding them into powder. DNA was extracted using a DNA extraction kit (Tiangen Biotech [Beijing] Co., Ltd.; DP34). The purity and concentration of the DNA were detected by electrophoresis using a 1% agarose gel (Tsingke Biotechnology Co., Ltd.; TSJ001).

The 16S rRNA sequencing process included polymerase chain reaction (PCR) amplification: Primers were designed for the V4 region of the 16S rRNA gene, with the primer sequences being 515F and 806R. To all PCR mixtures, 15 μL of Phusion^®^ High-Fidelity PCR Master Mix (New England Biolabs [NEB]; M0531S), 0.2 μM of primers, and 10 ng of genomic DNA template were added. An initial denaturation step was carried out at 98°C for 1 min, followed by 30 cycles of 98°C (10 s), 50°C (30 s), and 72°C (30 s), and a final extension at 72°C for 5 min. The PCR products were detected by electrophoresis using a 2% agarose gel. The qualified PCR products were purified using magnetic beads and quantified by enzyme-labeling. Samples were mixed in equal amounts according to the concentration of the PCR products. After thorough mixing, the PCR products were again detected by 2% agarose gel electrophoresis. The target bands were recovered using a universal DNA purification and recovery kit (Tiangen Biotech [Beijing] Co., Ltd.; DP214). Library construction was performed using the NEB Next® Ultra™ II FS DNA PCR-free Library Prep Kit (New England Biolabs [NEB]; E7430S). The constructed library was quantified by Qubit and Q-PCR. After the library passed the quality check, it was sequenced on an NovaSeq 6,000 platform with a PE 250 strategy (Callahan et al., 2019).

The data of each sample were split from the sequencing data according to the Barcode sequence and the PCR amplification primer sequence. After truncating the barcode and primer sequences, FLASH (Version 1.2.11) was used to splice the reads of each sample to obtain the raw Tags data (Raw Tags). The Cutadapt software was used to match the reverse primer sequence and cut the remaining sequence to prevent interference with subsequent analysis. The Fastp software (Version 0.23.1) was used to filter the obtained Raw Tags to obtain the Tags data (Clean Tags). The Tags sequences were aligned with the species annotation databases (Silva database https://www.arb-silva.de/ for 16S/18S, Unite database https://unite.ut.ee/for ITS) to remove chimeric sequences and obtain the final effective data (Effective Tags). The DADA2 module in the QIIME2 (Version QIIME2-202202) software was used on the Effective Tags to obtain the final amplicon sequence variants and the feature table. The QIIME2 software was used for species annotation with the Silva 138.1 database. For functional analysis, Tax4Fun (V0.3.1) was used to associate and map the sequences with the Kyoto Encyclopedia of Genes and Genomes (KEGG) database for predictive analysis of the functional genes and metabolic pathways of the microbial community (Bokulich et al., 2013; Edgar et al., 2011).

Analysis of similarities (ANOSIM) and multi-response permutation procedure (MRPP) were employed to assess microbial community differences. ANOSIM, based on ranks, compares between- and within-group differences (range −1 to 1). MRPP utilizes distance matrices and permutations, and the “A” value measures within-group cohesion (Koh et al., 2023). Additionally, Chao1 and Shannon indices were used. The Chao1 index estimates species richness, with a higher value indicating greater species abundance. The Shannon index comprehensively reflects species richness and evenness, and a larger value represents higher microbial community diversity.

### 2.7 Metabolomic analysis

Samples for metabolite detection: On a sterile operating bench, use a sterile spatula to scrape the metabolites from each intestinal segment, avoiding damage to the intestinal tissue. Place the samples in sterile 1.5 mL cryotubes, snap-freeze them in liquid nitrogen, and store them in a − 80°C refrigerator. The storage time should not exceed 10 days. Perform liquid chromatography-mass spectrometry as soon as possible to prevent metabolite degradation or spoilage.

Take out the samples from the −80°C freezer and thaw them on ice. The intestinal contents and blood samples are processed differently. For the intestinal content samples, take 100 mg of the sample, grind it with liquid nitrogen, add 500 μL of an 80% methanol aqueous solution, vortex and shake it, then let it stand in an ice bath for 5 min. Centrifuge it at 15,000 rpm at 4°C for 20 min. Take the supernatant, dilute it with mass spectrometry-grade water until the methanol content is 53%, centrifuge it again at 15,000 rpm at 4°C for 20 min, collect the supernatant, and inject it for liquid chromatography-mass spectrometry analysis. For the blood samples, take 100 μL and place it in an eppendorf tube, add 400 μL of an 80% methanol aqueous solution, and the remaining steps are the same as those for the intestinal content samples (Want et al., 2013).

*Quality control (QC) samples*: Take equal volumes from the experimental samples and mix them evenly.

Blank samples: Use a 53% methanol aqueous solution to replace the experimental samples, and the pretreatment process is the same.

Chromatographic conditions: Chromatographic column, Hypersil Gold column (C18) (Thermo Fisher Scientific); Column temperature, 40°C; Flow rate, 0.2 mL/min; Mobile phase A, 0.1% formic acid; Mobile phase B, methanol; Chromatographic gradient elution.

Mass spectrometry conditions (Thermo Fisher Scientific; Q Exactive™ HF): Scanning range, 100–1,500 m/z; Spray voltage, 3.5 kV; Sheath gas flow rate, 35 psi; Auxiliary gas flow rate, 10 L/min; Capillary temperature, 320°C; S-lens RF level, 60; Auxiliary gas heater temperature, 350°C; Polarity, positive, negative; MS/MS secondary scanning is data-dependent scans (Nemet et al., 2020).

The sequencing data are converted into mzXML format by ProteoWizard. XCMS is used for peak extraction and quantification, and peak alignment is carried out for different samples. Metabolites are identified according to the set mass deviation of 10 ppm, adduct ions, and a high-quality secondary spectrum database. Subsequently, use the Blank samples to remove the background ions.

Standardize the original quantitative results according to the formula: the original quantitative value of the sample/(the sum of the quantitative values of metabolites in the sample/the sum of the quantitative values of metabolites in the QC sample) to obtain the relative peak area. Delete the compounds with a relative peak area > 30% in the QC samples to obtain the final metabolite identification and relative quantification results (Yuan et al., 2012).

The data processing part is carried out using the Linux operating system (CentOS version 6.6), R, and Python. Use the KEGG database^1^ to perform functional annotation on the identified metabolites.

### 2.8 Statistical analysis methods

For data with normal distribution and homogeneous variance, paired t-tests were used for comparisons between two groups, while one-way analysis of variance with Tukey’s *post hoc* test was used for comparisons among multiple groups. For data that did not meet the normality assumption, non-parametric tests (Mann–Whitney U test or Kruskal–Wallis test) were applied. Statistical analysis was performed using GraphPad Prism 9 (GraphPad Software, San Diego, CA, United States), and data are expressed as mean ± standard deviation (mean ± SD). Significance was considered at *p* < 0.05.

## 3 Results

### 3.1 Alcohol-induced bone loss and increased intestinal permeability in mice

**Figure 1:**
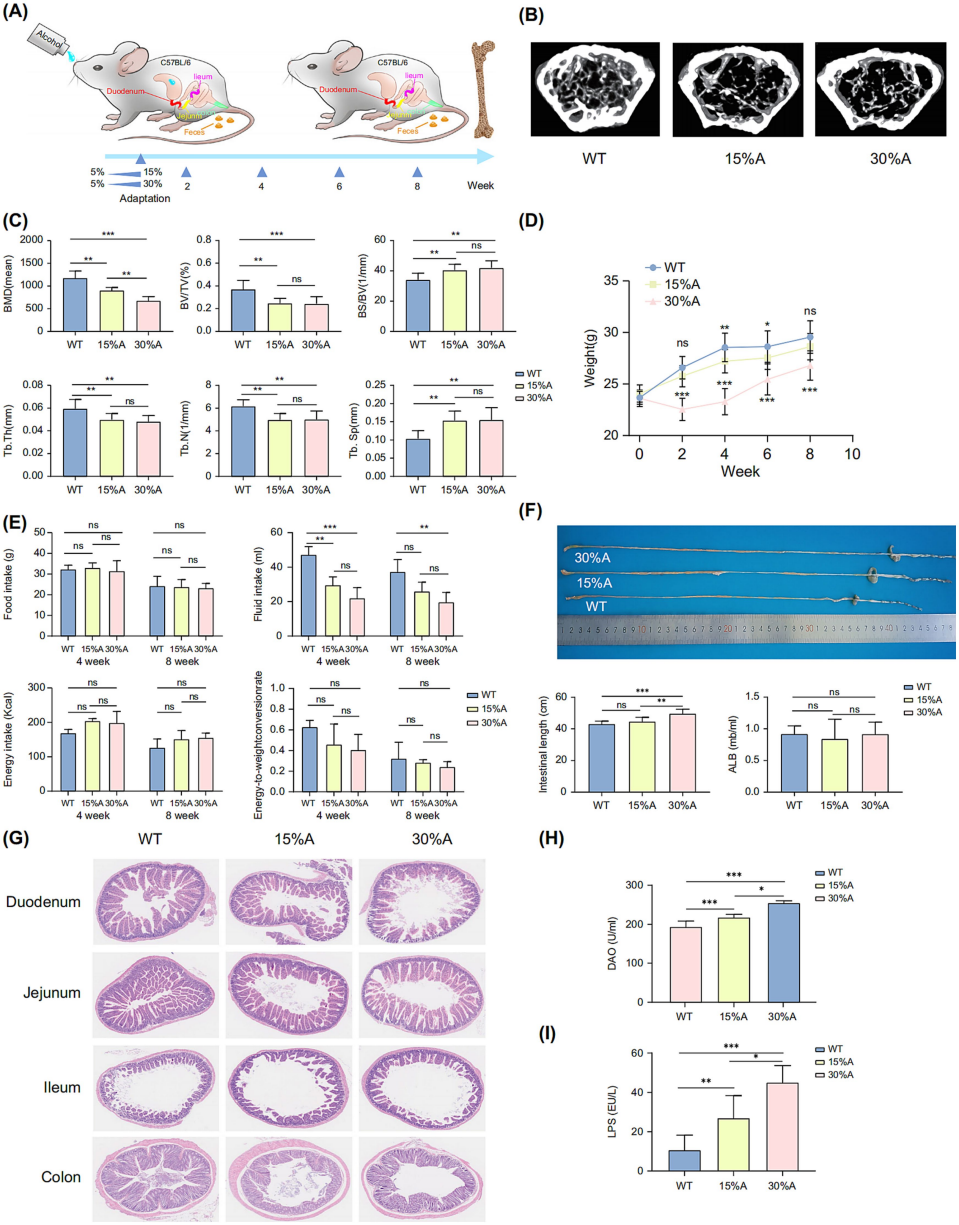
AOP model induction. **(A)** Illustration of the AOP model. **(B)** Femur micro-CT images (from left to right: WT, 15%-AOP, and 30%-AOP groups). **(C)** Bone parameter analysis: BMD, BV/TV, BS/BV, Tb.Th, Tb.N, and Tb.Sp. **(D)** Line graph of mouse body weight. **(E)** Liquid intake, food intake, total energy intake, and Bioconversion Efficiency. **(F)** Differences in total intestinal length and quantitative serum ALB levels. **(G)** H&E-stained histological images of different intestinal segments (duodenum, jejunum, ileum, colon). **(H)** Diamine Oxidase (DAO) serum levels. **(I)** Lipopolysaccharide (LPS) serum levels. Values in bar graphs represent means ± SDs. *p < 0.1, **p < 0.05, ***p < 0.01 (*n* = 6). ALB, albumin; AOP, alcohol-induced osteoporosis; BMD, bone mineral density; BS/BV, bone surface/bone volume; BV/TV, trabecular volume/tissue volume; CT, computed tomography; H&E, hematoxylin and eosin; SD, standard deviation; Tb.N, trabecular number; Tb.Sp, trabecular separation; Tb.Th, trabecular thickness; WT, wild type; ns, not significant.

The stability of the AOP mouse model (Figure 1A) was confirmed through microstructural observation of femoral trabeculae and bone density analysis. Compared to wild-type (WT) mice, alcohol-exposed mice exhibited significantly thinner, fewer, less dense, and more sparsely arranged trabeculae (Figure 1B). The AOP groups showed marked reductions in BMD, BV/TV, Tb.Th, and Tb.N, with corresponding increases in BS/BV and Tb.Sp compared to the WT group (*p* < 0.05). Higher alcohol concentrations exacerbated bone loss, with trabecular thickness showing a statistically significant reduction (*p* < 0.05; Figure 1C).

The 30%-AOP group consistently showed significantly lower body weights compared to the WT group (*p* < 0.01) (Crandall et al., 2015), followed by a gradual increase after 2 weeks. While the 15%-AOP group also had lower body weights than the WT group, no significant decline was observed, and the weights continued to increase over time (Figure 1D). No significant differences in total energy intake were observed between the experimental and control groups, and although the Energy-to-weight conversion rate was slightly lower in the experimental groups, this difference was not statistically significant (Figure 1E). Intestinal length analysis revealed a significant increase in the 30%-AOP group compared to the WT group (*p* < 0.01) (*p* < 0.05). ALB levels showed no significant differences between the experimental and WT groups (Figure 1F).

Histological results of different intestinal segments showed that although alcohol had no significant impact on the nutritional status of mice, it did cause damage to the intestines, particularly in the upper GI regions, such as the duodenum and jejunum (Figure 1G). This was supported by a significant increase in DAO levels (*p* < 0.01; Figure 1H) and LPS levels (*p* < 0.05; Figure 1I).

### 3.2 Microbiota in intestinal and feces show complex spatial distribution in normal mice

**Figure 2:**
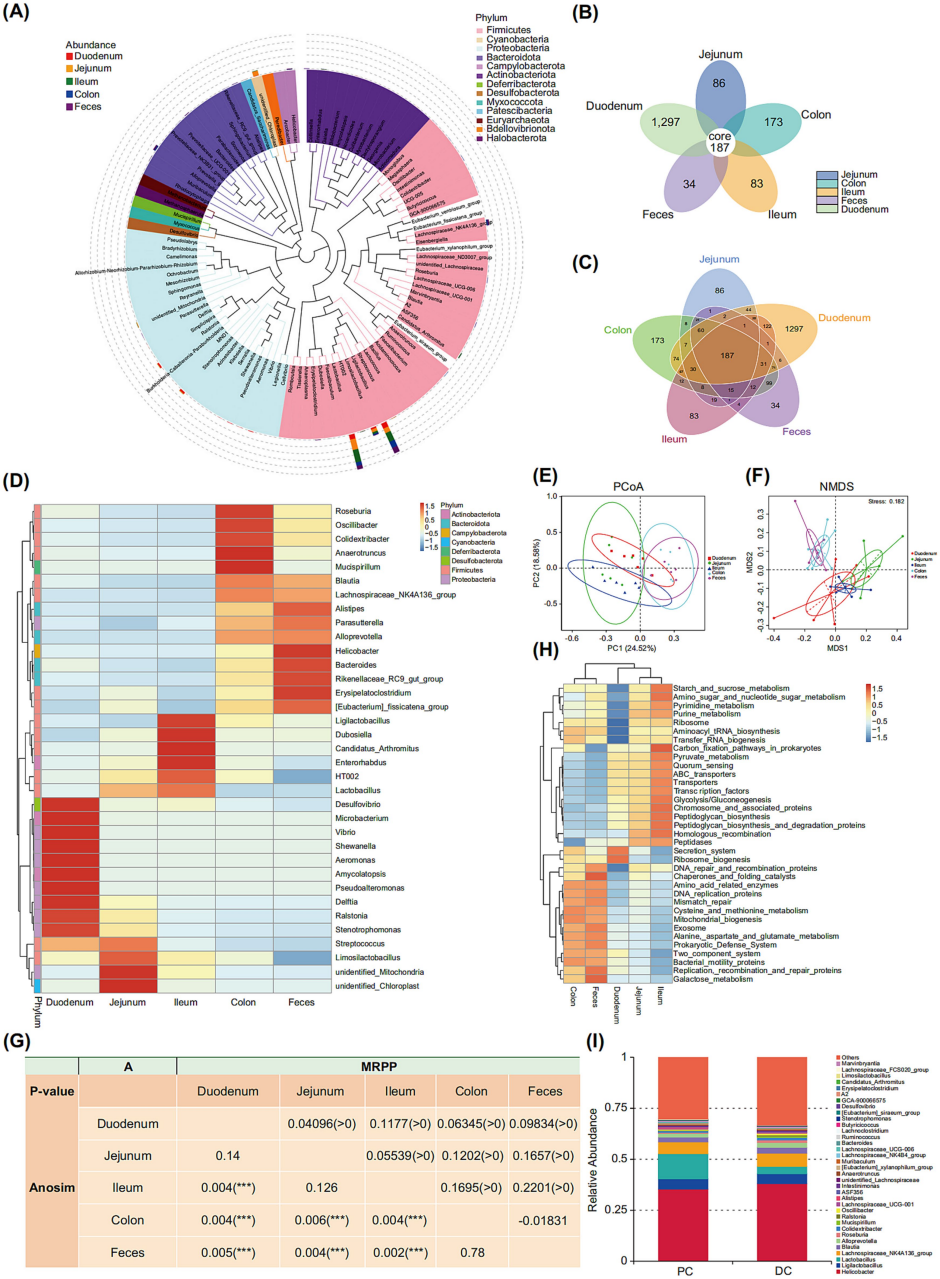
Analysis of 16S rRNA microbial communities in different intestinal segments and feces of normal mice. **(A)** Phylogenetic tree of microbial genera from different intestinal segments and feces. **(B)** Petal Diagram of microbial communities from different intestinal segments and feces. **(C)** Venn Diagram of microbial communities from different intestinal segments and feces. **(D)** Heatmap of microbial genera from different intestinal segments and feces. **(E)** Principal coordinates analysis (PCoA) plot of microbial communities from different intestinal segments and feces. **(F)** Non-Metric Multidimensional Scaling (NMDS) plot of microbial communities from different intestinal segments and feces. **(G)** Multi-response permutation procedure (MRPP) and analysis of similarities (ANOSIM) analyses of microbial communities from different intestinal segments and feces, **(H)** Heatmap of KEGG functional profiles of microbial communities from different intestinal segments and feces, **(I)** Bar chart of microbial communities in the proximal and distal colon. Values in the bar graphs represent means ± SDs. *p < 0.1, **p < 0.05, ***p < 0.01 (*n* = 6). DC, Distal Colon; PC, Proximal Colon.

A genus-level phylogenetic analysis of the microbiota across different intestinal segments and feces of AOP-model mice was conducted, resulting in a clear demarcation of the evolutionary differences in microbial composition among these regions. For instance, most genera found in all intestinal regions and feces were classified under the phylum Firmicutes, including *Ligilactobacillus*, *Lactobacillus*, and *Limosilactobacillus*. Moreover, significant differences in the evolutionary origins of microbiota between the upper and lower GI tracts were observed. The microbiota in the upper GI tract (duodenum and jejunum) was predominantly composed of genera from the phylum Proteobacteria, such as *Ralstonia* and *Stenotrophomonas*. In contrast, the lower GI tract (colon and feces) was primarily characterized by genera from the phylum Firmicutes (e.g.*, Lachnospiraceae_NK4A136_group*) and the phylum Bacteroidota (e.g.*, Alloprevotella*). Interestingly, although certain phylogenetic branches in the jejunum displayed higher abundance, many of the genera remain undefined, highlighting significant gaps in our understanding of upper GI tract microbiota (Figure 2A).

An analysis of the spatial distribution differences in the shared microbiota of mice showed that the unique Operational Taxonomic Units (OTUs) in the duodenum, jejunum, ileum, colon, and feces accounted for 69.7, 4.6, 4.5, 9.3, and 1.8% of the total OTUs, respectively, while the 187 OTUs shared among these five groups represented 10.1% of the total 1,860 OTUs (Figures 2B,C).

Overall, the abundance of Firmicutes in the ileum was significantly higher than in all other intestinal segments and feces, except for the jejunum (*p* < 0.01). Conversely, the abundance of Bacteroidota was significantly higher in the colon and feces compared to other segments (*p* < 0.01). Notable differences were also observed between adjacent segments. For instance, Patescibacteria was significantly more abundant in the jejunum than in the duodenum (*p* < 0.05), while Actinobacteriota in the colon showed significantly higher abundance compared to feces (*p* < 0.05; Table 1; Supplementary Figure S1). At the genus level, Streptococcus in the duodenum was significantly more abundant than in feces (*p* < 0.05). *Lactobacillus* in the ileum showed significantly higher abundance compared to all other intestinal segments and feces, except for the jejunum (*p* < 0.05). *Candidatus Arthromitus* was more abundant in all intestinal segments than in feces (*p* < 0.05), and *Aistipes* and *Anaerotruncus* in the colon showed significantly higher abundance compared to all other segments except feces (*p* < 0.05;Table 2). These differences were identified based on an analysis limited to taxa with mean abundances greater than 0.001 in all groups (Supplementary Figure S2). Additionally, the duodenum contained certain unique taxa that were scarcely present in other intestinal segments or feces, even at the phylum or genus level. These taxa included Halobacterota, Cloacimonadota, *Pseudoalteromonas*, *Aeromonas*, and *Shewanella* (Figure 2D). Next, using principal coordinates analysis (PCoA) (Figure 2E) and Non-Metric Multidimensional Scaling (NMDS) (Figure 2F) plots allow visualization of significant differences in genus-level diversity between the upper and lower GI tracts.

**Table 1:** Dominant microbial flora at the phylum level in different intestinal segments and feces of normal mice.

Dominant microbial flora (phylum level)					
	Duodenum	Jejunum	Ileum	Colon	Feces
Duodenum	–	–	–	–	–
Jejunum	Patescibacteria (*p* = 0.011)	–	–	–	Actinobacteriota (*p* = 0.03)
Ileum	Firmicutes (*p* < 0.001)	–	–	Firmicutes (*p* < 0.001) Proteobacteria (*p* = 0.039)	Firmicutes (*p* < 0.001) Actinobacteriota (*p* = 0.03) Proteobacteria (*p* = 0.049)
Colon	Bacteroidota (*p* < 0.001)	Bacteroidota (*p* < 0.001)	Bacteroidota (*p* < 0.001)	–	Actinobacteriota (*p* = 0.027)
Feces	Bacteroidota (*p* < 0.001)	Bacteroidota (*p* < 0.001)	Bacteroidota (*p* < 0.001)	–	–

**Table 2:** Dominant microbial flora at the genus level in different intestinal segments and feces of normal mice.

Dominant microbial flora (genus level)					
	Duodenum	Jejunum	Ileum	Colon	Feces
Duodenum	–				Streptococcus (*p* = 0.045)
Jejunum	Candidatus_Saccharimonas (*p* = 0.01)	–	–	–	*Limosilactobacillus* (*p* = 0.022) Enterorhabdus (*p* = 0.049)
Ileum	*Lactobacillus* (*p* = 0.023) Candidatus_Arthromitus (*p* = 0.017) Enterorhabdus (*p* = 0.049)	Candidatus_Arthromitus (*p* = 0.02)	–	*Lactobacillus* (*p* = 0.008)Candidatus_Arthromitus (*p* = 0.021) Ralstonia (*p* = 0.048)	*Lactobacillus* (*p* = 0.008)Candidatus_Arthromitus (*p* = 0.019) Enterorhabdus (*p* = 0.036) Ralstonia (*p* = 0.043)*Limosilactobacillus* (*p* = 0.035)
Colon	Candidatus_Arthromitus (*p* = 0.001) Prevotellaceae_UCG-001 (*p* = 0.021) *Muribaculum* (*p* = 0.024) *Anaerotruncus* (*p* = 0.045) Alistipes (*p* = 0.047)	*Muribaculum* (*p* = 0.013)Prevotellaceae_UCG-001 (*p* = 0.02) *Anaerotruncus* (*p* = 0.038) Alistipes (*p* = 0.039)	*Bacteroides* (*p* = 0.046) Alistipes (*p* = 0.04) *Anaerotruncus* (*p* = 0.038) Prevotellaceae_UCG-001 (*p* = 0.02)	–	Enterorhabdus (*p* = 0.03)
Feces	*Muribaculum* (*p* = 0.005)Prevotellaceae_UCG-001 (*p* = 0.017) Alistipes (*p* = 0.029) *Alloprevotella* (*p* = 0.045)	*Muribaculum* (*p* = 0.004)Prevotellaceae_UCG-001 (*p* = 0.017) Alistipes (*p* = 0.025) *Bacteroides* (*p* = 0.038) *Alloprevotella* (*p* = 0.044)	Prevotellaceae_UCG-001 (*p* = 0.017) Alistipes (*p* = 0.026) *Bacteroides* (*p* = 0.032) *Alloprevotella* (*p* = 0.045)	–	–

ANOSIM and MRPP analysis confirmed significant differences in microbial communities across various intestinal segments and feces, particularly between the upper and lower digestive tracts (*p* < 0.01). Significant differences were also observed among the duodenum, jejunum, and ileum (*A* > 0), whereas no notable differences were observed between the microbial communities of the lower GI tract (e.g., colon) and fecal flora (Figure 2G).

Overall, the microbiota in the upper GI tract (e.g., duodenum and jejunum) showed significant involvement in functions related to transporters, pyruvate metabolism, and glycolysis/gluconeogenesis compared to the lower GI tract microbiota (*p* < 0.05). Specific examples included adenylate kinase, the phosphoenolpyruvate-sugar phosphotransferase system, glucose-specific IIA component, and aspartate aminotransferase (*p* < 0.05). In contrast, the microbiota in the lower GI tract (e.g., colon and feces) demonstrated greater prominence in functions related to amino acid-related enzymes and mitochondrial biogenesis (*p* < 0.01). Examples include dipeptidase, RNA polymerase sigma factor, and ATP-binding cassette transporter (*p* < 0.05; Figure 2H and Supplementary Figure S3). When analyzing specific segments of the gut, no significant involvement in functions were observed between the colonic and fecal microbiota. Similarly, the microbiota in the duodenum and jejunum exhibited minimal involvement in functions, with only the longevity-regulating pathway and prenyltransferases showing significant variation (*p* < 0.05). For instance, glutamate dehydrogenase, NAD(P) + was one of the enzymes displaying such differences (*p* < 0.05; Supplementary Figure S4).

Since the colonic microbiota exhibits the highest abundance across all intestinal segments, we further investigated whether there are differences between the proximal colon and the distal colon microbiota (Bergstrom et al., 2020). Multiple levels of analysis revealed no significant differences in diversity or abundance between the two regions (Figure 2I). However, in terms of microbial functions, notable differences were observed: the proximal colon showed higher levels of lysyl-transfer RNA synthetase and sulfatase (*p* < 0.05), while the distal colon demonstrated significantly elevated expression of ornithine decarboxylase and lactase (*p* < 0.05; Supplementary Figure S5).

### 3.3 Alcohol causes significant dysbiosis of microbiota in different intestinal and feces of mice

**Figure 3:**
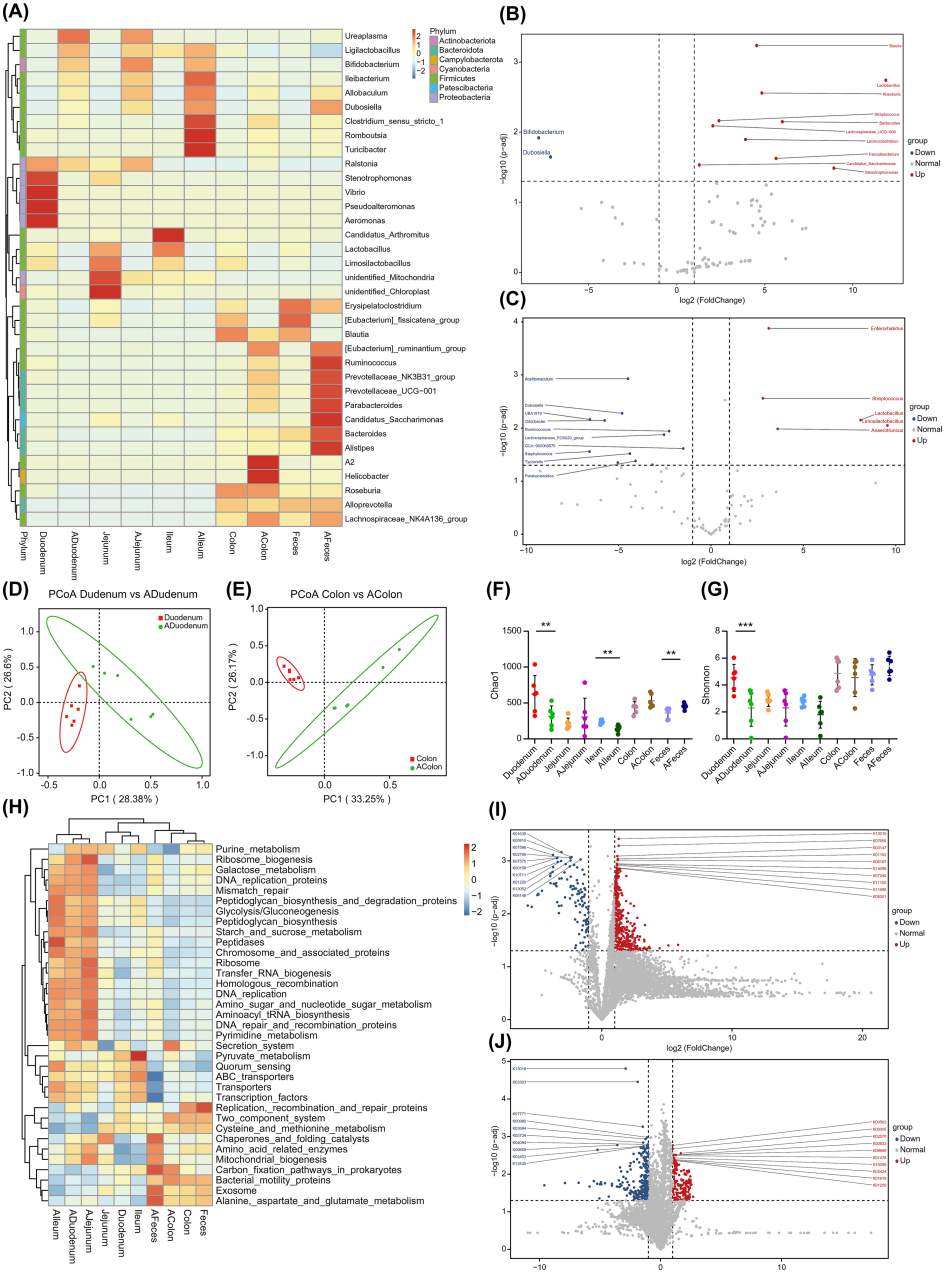
Alcohol-induced differential microbial changes in various intestinal segments and feces of mice. **(A)** Heatmap of genus-level abundance clustering in different intestinal segments and feces. **(B)** Volcano plot of metastat analysis for the duodenum. **(C)** Volcano plot of metastat analysis for the colon. **(D)** PCoA analyses of the duodenum. **(E)** PCoA analyses of the colon. **(F)** Alpha diversity analysis: Chao1 index. **(G)** Alpha diversity analysis: Shannon diversity index. **(H)** Tax4Fun functional prediction volcano plot for the duodenum. **(I)** Volcano plot of KEGG analysis for the duodenum. **(J)** Volcano plot of KEGG analysis for the colon. Values in the bar graphs represent means ± SDs. *p < 0.1, **p < 0.05, ***p < 0.01 (*n* = 6). KEGG, Kyoto Encyclopedia of Genes and Genomes; NMDS, non-metric multidimensional scaling; PCoA, principal coordinate analysis; SD, standard deviation.

A comparative analysis of gut microbiota changes across different intestinal regions and feces after alcohol consumption showed that at the phylum level, the Campylobacterota and Patescibacteria in the duodenum showed significant reductions, while Actinobacteriota exhibited a marked increase. In the jejunum, both Patescibacteria and Cyanobacteria were significantly elevated, accompanied by a notable rise in Actinobacteriota. In the ileum, only Actinobacteriota showed a significant increase. In contrast to the changes observed in the duodenum, the colon showed a significant increase in Campylobacterota alone. In feces, both Patescibacteria and Actinobacteriota were significantly elevated (*p* < 0.05; Figure 3A; Table 3).

**Table 3:** Effects of alcohol on the bacterial at the phylum level in different intestinal segments and feces.

	Up	Down
Duodenum	Actinobacteriota (*p* = 0.046)	Campylobacterota (*p* = 0.02) Patescibacteria (*p* = 0.043)
Jejunum	Actinobacteriota (*p* < 0.01)	Patescibacteria (*p* < 0.01) Cyanobacteria (*p* = 0.044)
Ileum	Actinobacteriota (*p* = 0.02)	Firmicutes (*p* = 0.049)
Colon	Campylobacterota (*p* = 0.025)	Bacteroidota (*p* = 0.018)
Feces	Actinobacteriota (*p* < 0.01) Patescibacteria (*p* < 0.01)	–

At the genus level, the duodenum exhibited significant reductions in *Candidatus Saccharimonas*, *Lactobacillus*, and *Roseburia* (Figure 3B). Similarly, the jejunum showed notable decreases in *Candidatus Saccharimonas*, *Lactobacillus*, and *Limosilactobacillus*, while *Muribaculum* and *Turicibacter* were significantly increased. In the ileum, genera such as Lactobacillus, *Candidatus Saccharimonas*, *Limosilactobacillus*, and *Bacteroides* experienced significant reductions, while *Faecalibaculum* and *Allobaculum* showed notable increases. In the colon, *Lactobacillus* and *Limosilactobacillus* were significantly reduced, whereas *Dubosiella*, *Acetitomaculum*, and *Ruminococcus* were markedly elevated (Figure 3C). In feces, *Anaerofustis* and *Anaerotruncus* experienced significant reductions, while *Bifidobacterium*, *Candidatus Saccharimonas*, and *Odoribacter* significantly increased. Additionally, *Dubosiella* and *Bifidobacterium* showed a notable upward trend in the duodenum, jejunum, and ileum (*p* < 0.05; Table 4; Supplementary Figure S6).

**Table 4:** Effects of alcohol on the bacterial at the genus level in different intestinal segments and feces.

	Up	Down
Duodenum	Bifidobacterium (*p* = 0.012) Dubosiella (*p* = 0.023)	*Lactobacillus* (*p* < 0.01); *Bacteroides* (*p* < 0.01) Roseburia (*p* < 0.01); Blautia (*p* < 0.01) Streptococcus (*p* < 0.01); Lachnospiraceae_UCG-006 (*p* < 0.01); Lachnoclostridium (*p* = 0.012); Faecalibacterium (*p* = 0.024); Candidatus_Saccharimonas (*p* = 0.03) Stenotrophomonas (*p* = 0.032)
Jejunum	Bifidobacterium (*p* < 0.01); Turicibacter (*p* = 0.04) *Muribaculum* (*p* = 0.013); Dubosiella (*p* = 0.015)[Eubacterium]_ruminantium_group (*p* = 0.038)	*Lactobacillus* (*p* < 0.01); *Limosilactobacillus* (*p* < 0.01); Candidatus_Saccharimonas (*p* < 0.01); Anaerofustis (*p* < 0.01); [Eubacterium]_brachy_group (*p* < 0.01) Enterorhabdus (*p* = 0.036); Parvibacter (*p* = 0.042)
Ileum	Bifidobacterium (*p* < 0.01); Dubosiella (*p* < 0.01) *Faecalibaculum* (*p* < 0.01); Allobaculum (*p* = 0.018)	*Lactobacillus* (*p* < 0.01); Ralstonia (*p* < 0.01) *Limosilactobacillus* (*p* < 0.01); Enterorhabdus ( *p* < 0.01) Parvibacter (*p* = 0.04); *Bacteroides* (*p* = 0.018) Candidatus_Saccharimonas (*p* = 0.028); Streptococcus (*p* = 0.029) [Eubacterium]_brachy_group (*p* = 0.043)
Colon	Dubosiella (*p* < 0.01); Odoribacter (*p* < 0.01) Acetitomaculum (*p* < 0.01); Ruminococcus (*p* = 0.011) Lachnospiraceae_FCS020_group (*p* = 0.013) Staphylococcus (*p* = 0.028); Tyzzerella (*p* = 0.03) Parabacteroides (*p* = 0.042); Helicobacter (*p* = 0.045)	*Lactobacillus* (*p* < 0.01); *Limosilactobacillus* (*p* < 0.01) Streptococcus (*p* < 0.01); Enterorhabdus (*p* < 0.01) *Anaerotruncus* (*p* < 0.01)
Feces	Bifidobacterium (*p* = 0.015); Alistipes (*p* = 0.03) Parabacteroides (*p* = 0.032); Candidatus_Saccharimonas (*p* < 0.01) *Faecalibaculum* (*p* = 0.024); Anaerovorax (*p* < 0.01) Lachnospiraceae_FCS020_group (*p* < 0.01) NK4A214_group (*p* < 0.01) Odoribacter (*p* < 0.01); Candidatus_Soleaferrea (*p* = 0.026)	*Anaerotruncus* (*p* = 0.024) Anaerofustis (*p* < 0.01)

Overall, microbial changes in different intestinal regions and feces exhibit significant regional differences (Figures 3D,E), particularly resulting in a decrease in beneficial genera in the upper GI tract and an increase in pathogenic bacteria in the lower GI tract. The Chao1 index (Figure 3F) significantly decreased in the duodenum and jejunum (*p* < 0.05) but significantly increased regarding fecal microbiota (*p* < 0.05). Correspondingly, the Shannon index (Figure 3G) showed a significant decrease in the duodenum (*p* < 0.01). These findings suggest that alcohol consumption significantly disrupts the previously stable spatial distribution of gut and fecal microbiota, while also causing notable changes in microbial diversity.

When exploring various diseases caused by changes in the intestinal environment, it is important to conduct in-depth research and analysis on the gut microbiota. Different alterations in the intestinal environment can cause the gut microbiota to exhibit unique characteristics. Using differential research, we can accurately identify the characteristic taxonomic microbiota unique to different environments, which provides crucial clues for subsequent scientific research exploration. Alcohol is one of many factors that affect the gut microbiota. Peritoneal dialysis modifies the extra-intestinal environment and can significantly change the composition and structure of the gut microbiota in patients (Fan et al., 2024). This phenomenon fully demonstrates that comprehensive research on the gut microbiota is of great importance and improves our understanding of the complex relationship between diseases and the gut microbiota.

Functional predictions based on Tax4Fun indicated that alcohol significantly increased the activity of transfer RNA biogenesis, pyrimidine metabolism, glycolysis/gluconeogenesis, chromosome associated proteins, and peptidases in the upper GI tract. In the lower GI tract, pathways such as carbon fixation pathways in prokaryotes, butanoate metabolism, ribosome and mitochondrial biogenesis, and chaperones and folding catalysts also showed abnormally elevated activity. Conversely, alcohol suppresses key functions in the upper GI tract, including the two-component system, carbon fixation pathways in prokaryotes, cysteine and methionine metabolism, and glycine serine and threonine metabolism. Similarly, in the lower GI tract, ethanol inhibited pathways such as purine metabolism, cysteine and methionine metabolism, galactose metabolism, the two-component system, and replication, recombination, and repair proteins (Figure 3H and Supplementary Figure S7). Notably, alcohol significantly enhanced the expression of bacterial flagellar assembly, a function closely associated with the microbiota, in the duodenum (Supplementary Figure S8). In terms of the number of functional impacts on the microbiota, alcohol had a significant effect on the lower GI tract, particularly on feces (Figures 3I,J and Supplementary Figure S9).

### 3.4 Intestinal metabolite differences in AOP mice

**Figure 4:**
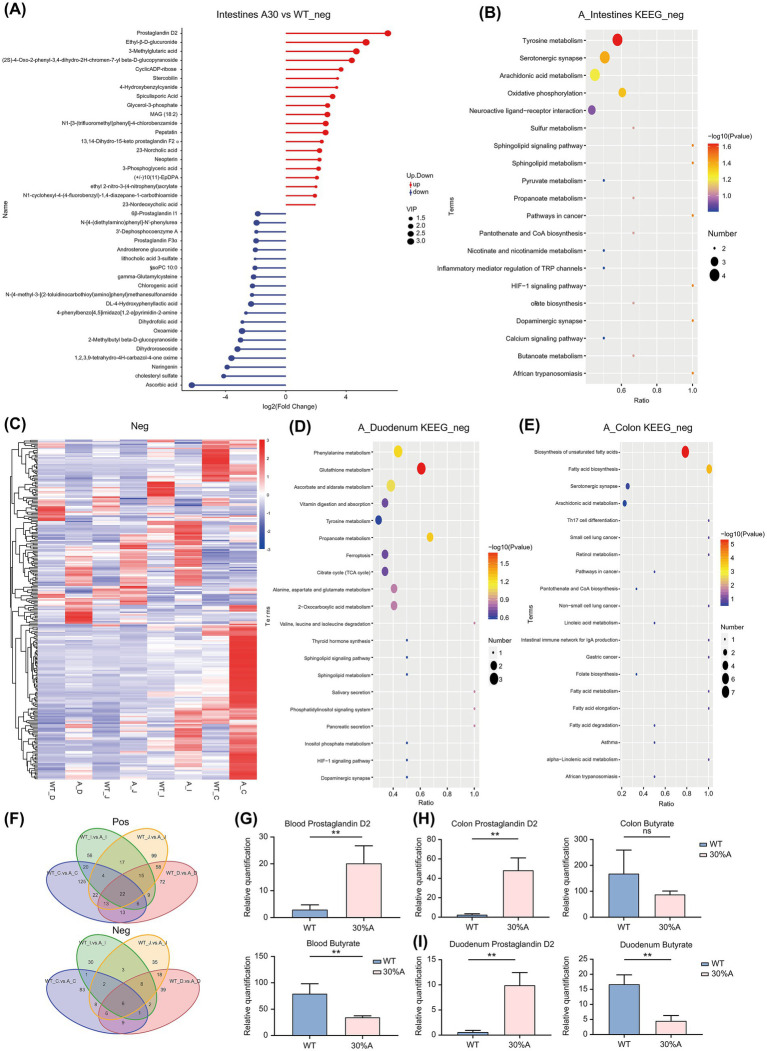
Intestinal Metabolite Differences in Alcoholic Osteoporosis Mice. **(A)** Matchstick graph for differential metabolites across the entire intestine. **(B)** Kyoto Encyclopedia of Genes and Genomes (KEGG) pathway enrichment map of NEG metabolites across the entire intestine. **(C)** Differential heatmap of NEG metabolites in different intestinal segments. **(D)** KEGG pathway enrichment map of NEG metabolites in the duodenum. **(E)** KEGG pathway enrichment map of NEG metabolites in the colon. **(F)** Venn diagram of metabolites in different intestinal segments. **(G)** Relative amount of PGD2 and butyrate in the blood. **(H)** Relative quantification of PGD2 and butyrate in the colon. **(I)** Relative quantification of PGD2 and butyrate in the duodenum. Values in bar graphs represent means ± SDs. *p < 0.1, **p < 0.05, ***p < 0.01 (*n* = 6). NEG, negative; PGD2, prostaglandin D2; SD, standard deviation.

Metabolite analysis of both positive and negative ions across the entire intestines of AOP mice revealed significant changes. Notable elevations were observed in metabolites such as PGD2, ethyl-*β*-D-glucuronide, 3-methylglutaric acid, ellagic acid, and spermine. Conversely, the levels of ascorbic acid, cholesteryl sulfate, naringenin, norsufentanil, L-ascorbate, and homotaurine were significantly reduced. KEGG pathway enrichment analysis indicated that alcohol consumption caused notable alterations in cellular pathways, including tyrosine metabolism, serotonergic synapse, oxidative phosphorylation, cysteine and methionine metabolism, synaptic vesicle cycle, and sulfur relay system (*p* < 0.05; Figures 4A,B and Supplementary Figure S10).

Further metabolite analysis revealed significant variations across intestinal segments and feces of AOP mice. Ethyl-β-D-glucuronide and PGD2 were notably elevated in multiple segments, including the jejunum, ileum, and colon. Other elevated metabolites included cyclic ADP-ribose and psilocybin in the duodenum, glycerol-3-phosphate and docosatrienoic acid in the colon, as well as 13,14-dihydro prostaglandin F1α and N1-isopropyl-2-(phenylthio)benzamide in feces. Conversely, ascorbic acid and cysteinylglycine showed significant reductions in both the duodenum and jejunum. Additionally, decreases were observed in 3′-dephosphocoenzyme A and chlorogenic acid in the duodenum, cholesteryl sulfate and naringenin chalcone in the colon, and naringenin and mupirocin in feces (*p* < 0.05; Figure 4C and Supplementary Figures S11–S14). This integrated analysis highlights consistent changes in certain metabolites, such as PGD2, ethyl-β-D-glucuronide, and ascorbic acid, across multiple sites, suggesting systemic and localized impacts of alcohol consumption on intestinal metabolism.

In the KEGG analysis of local metabolite functions, significant differences were observed in the metabolic pathways across various intestinal segments and feces. Specifically, glutathione metabolism and synaptic vesicle cycle showed notable changes in the duodenum, jejunum, and feces. Additionally, the duodenum exhibited changes in propanoate metabolism, sulfur relay system, and Inflammatory mediator regulation of transient receptor potential channels, while the jejunum included inflammatory mediator regulation of transient receptor potential channels and purine metabolism. In the ileum, significant alterations were identified in sphingolipid signaling pathway, sphingolipid metabolism, and metabolism of xenobiotics by cytochrome P450. The colon displayed notable differences in biosynthesis of unsaturated fatty acids, tryptophan metabolism, and retinol metabolism, whereas feces showed significant changes in protein digestion and absorption and mineral absorption. These findings suggest that the prominent changes in glutathione metabolism and synaptic vesicle cycle may play a key role in systemic regulation (*p* < 0.05; Supplementary Figure S15).

Overall, compared to the distinct differences in gut microbiota observed between the upper and lower digestive tracts induced by alcohol consumption, the distribution of gut metabolites across different intestinal segments exhibited a relatively similar variation trend (Figure 4F). This may be attributed to the mobility of metabolites. However, the functional roles of metabolites still differ across regions, further emphasizing the spatial complexity and significant local variability of alcohol’s impact on gut microbiota and metabolites.

Relative quantitative detection of PGD2 in the blood revealed a significant increase in the AOP group (*p* < 0.01), suggesting that changes in gut metabolites may induce systemic effects. PGD2 is recognized as an inflammatory mediator. Therefore, we also measured butyrate levels, given its reported anti-inflammatory properties in previous studies, and found that butyrate levels in the blood of the AOP group were significantly reduced (*p* < 0.01; Figure 4G). Additionally, PGD2 levels in the duodenum (Figure 4H) and colon (Figure 4I) of AOP mice were significantly elevated (*p* < 0.01). In contrast, butyrate levels were significantly reduced in the duodenum (*p* < 0.01) and showed a decreasing trend in the colon, although not statistically significant.

### 3.5 *Roseburia intestinalis* exhibits therapeutic effects on alcohol-induced Bone loss

**Figure 5:**
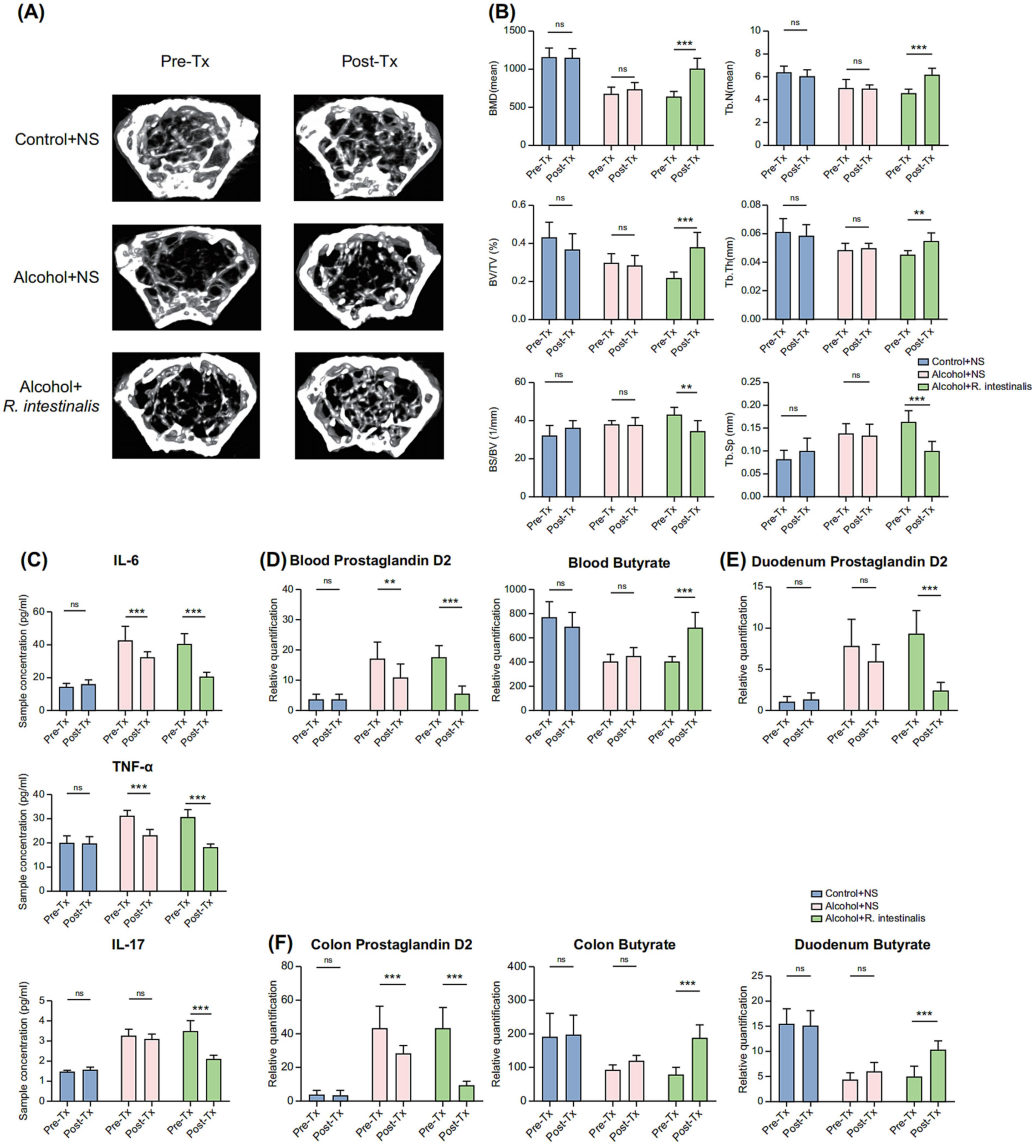
Therapeutic effect of *Roseburia intestinalis* on alcohol-induced bone loss. **(A)** Micro-CT images of mice femur post-treatment with *R. intestinalis*. **(B)** Bone parameter analysis: BMD, BV/TV, BS/BV, Tb.Th, Tb.N, and Tb.Sp. **(C)** Blood inflammatory factors IL-6, TNF-α, and IL-17. **(D)** Relative amounts of PGD2 and butyrate in the blood; **(E)** Relative quantification of PGD2 and butyrate in the duodenum. **(F)** Relative quantification of PGD2 and butyrate in the colon. Values are presented in graphs as means ± SDs. *p < 0.1, **p < 0.05, ***p < 0.01 (*n* = 6). BMD, bone mineral density; BS/BV, bone surface/bone volume; BV/TV, trabecular volume/tissue volume; CT, computed tomography; IL, interleukin; PGD2, prostaglandin D2; SD, standard deviation; Tb.N, trabecular number; Tb.Sp, trabecular separation; Tb.Th, trabecular thickness; TNF, tumor necrosis factor; Tx, Treatment; NS, Normal saline.

Although *R. intestinalis* is not directly associated with alcohol-induced bone loss, it produces butyrate, which has been shown to influence bone metabolism (Kang et al., 2023). In AOP mice, the abundance of *R. intestinalis* in the duodenum significantly decreased (*p* < 0.05), while no significant changes were observed in the colon (Supplementary Figure S16). To evaluate the therapeutic role of alcohol-depleted *R. intestinalis* in AOP, the well-characterized bacterium *R. intestinalis* was selected for supplementation experiments. After treatment, mice receiving *R. intestinalis* demonstrated significant improvements in BMD, BV/TV, Tb.N (*p* < 0.01), and Tb.Th (*p* < 0.05). Additionally, reductions in BS/BV and Tb.Sp (*p* < 0.01) were observed. These improvements were markedly greater compared to untreated AOP mice (Figures 5A,B).

Supplementation with *R. intestinalis* significantly reduced serum levels of IL-6, TNF-*α*, and IL-17 (*p* < 0.01; Figure 5C). Following treatment, serum butyrate levels increased significantly (*p* < 0.01), while PGD2 levels were notably suppressed (*p* < 0.01; Figure 5D). Quantitative analyses further confirmed that *R. intestinalis* significantly reduced alcohol-induced increases in PGD2 levels in both the duodenum and colon, while enhancing butyrate production in these regions (*p* < 0.01; Figures 5E,F).

Presently, the global aging process is accelerating continuously and the number of people suffering from osteoporosis is gradually increasing with a growing variety of treatment methods. In our research, we used *R. intestinalis* to treat osteoporosis by enhancing butyrate to inhibit the activation of IL-17 mediated by abnormally elevated PGD2, thereby improving the condition. Other effective treatments include using nanovesicles derived from *Rhizoma drynariae* to reverse osteoporosis by targeting the estrogen receptor α signaling pathway to enhance the osteogenic differentiation of human bone marrow mesenchymal stem cells (Zhao et al., 2024).

Considering that food-source factors, such as alcohol, can disrupt the gut microbiota and trigger osteoporosis, we can consider using natural medicinal herbs or their extracted active ingredients to protect the intestinal barrier, inhibit inflammatory factors (Li et al., 2025), block the progression of bone loss in the gut-bone axis pathway, and provide new ideas for osteoporosis treatment.

### 3.6 Alcohol induces an increase in the systemic Th17/Treg cell ratio and promotes osteoclast proliferation in mice

**Figure 6:**
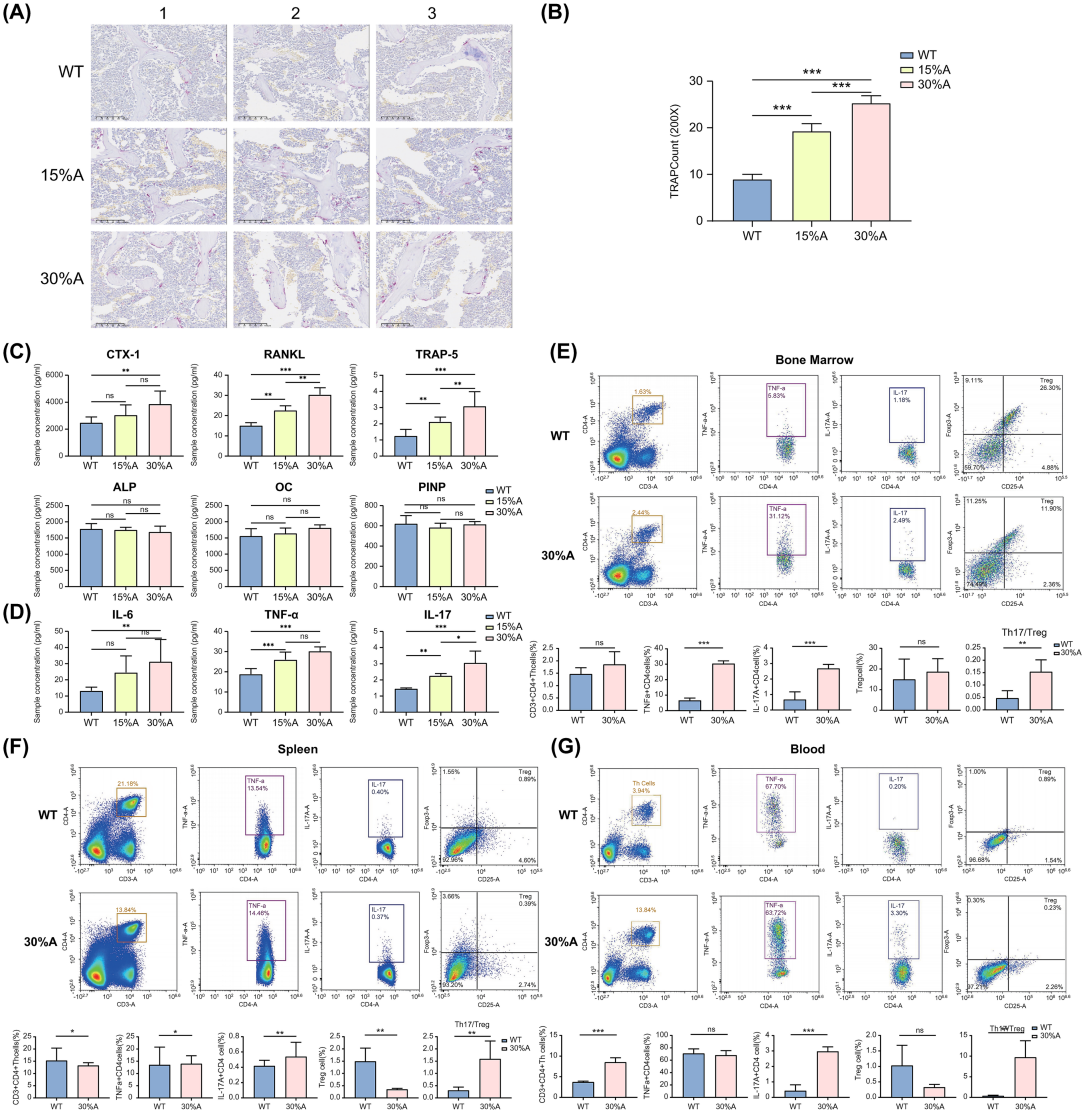
Increased Osteoclasts and Th17 Cell Populations in the Femurs of AOP Mice. **(A)** TRAP staining images under 20× magnification: from top to bottom, WT, 15%-AOP, and 30%-AOP groups. **(B)** Analysis of osteoclast numbers under 20× magnification. **(C)** Bone resorption markers (CTX-1, TRAP-5b, and RANKL) and bone formation markers (ALP, OC, and P1NP). **(D)** Inflammation-related factors: IL-6, TNF-α, and IL-17. **(E)** Blood flow cytometry analysis and differences. **(F)** Spleen flow cytometry analysis and differences. **(G)** Bone marrow flow cytometry and differential analysis. Values in the bar graphs represent means ± SDs. *p < 0.1, **p < 0.05, ***p < 0.01 (*n* = 6). ALP, alkaline phosphatase; AOP, alcohol-induced osteoporosis; CTX-1, C-terminal telopeptide of type I collagen; IL, interleukin; OC, osteocalcin; P1NP, procollagen type I N-terminal propeptide; RANKL, receptor activator of nuclear factor kappa beta ligand; SD, standard deviation; TNF, tumor necrosis factor; TRAP, tartrate-resistant acid phosphatase; WT, wild type.

TRAP staining of femurs revealed a significant increase in osteoclast numbers in both the 15%- and 30%-AOP groups compared to the WT group (*p* < 0.01; Figures 6A,B). The serum levels of TRAP-5b, RANKL (*p* < 0.01), and CTX-1 (*p* < 0.05), associated with osteoclast activation, were significantly elevated in the AOP groups. No significant differences were observed in osteoblast-differentiation markers, including OC, ALP, and P1NP (Figure 6C). Furthermore, serum levels of inflammation-related factors–IL-6 (*p* < 0.05), IL-17 (*p* < 0.01), and TNF-*α* (*p* < 0.01)–significantly increased in the 30%-AOP group compared to the WT group (Yu et al., 2021) (Figure 6D).

To further understand the immune mechanisms underlying bone loss, we compared the levels of immune-related cells in the blood, spleen, and bone marrow between the WT and 30%-AOP groups. The results indicated that the proportions of TNFα+CD4 + Th (p < 0.01) and IL-17A + CD4 Th (*p* < 0.01) cells (Guo et al., 2023) in the bone marrow were significantly higher in the 30%-AOP group leading to a significant increase in the Th17/Treg ratio (*p* < 0.05), which more accurately reflects Th17 cells function (Figure 6E). Similar trends were observed in the spleen (Figure 6F) and blood samples (Figure 6G).

### 3.7 Inhibiting PGD2 can alleviate alcohol-induced bone loss by reducing the Th17/Treg ratio in the blood

**Figure 7:**
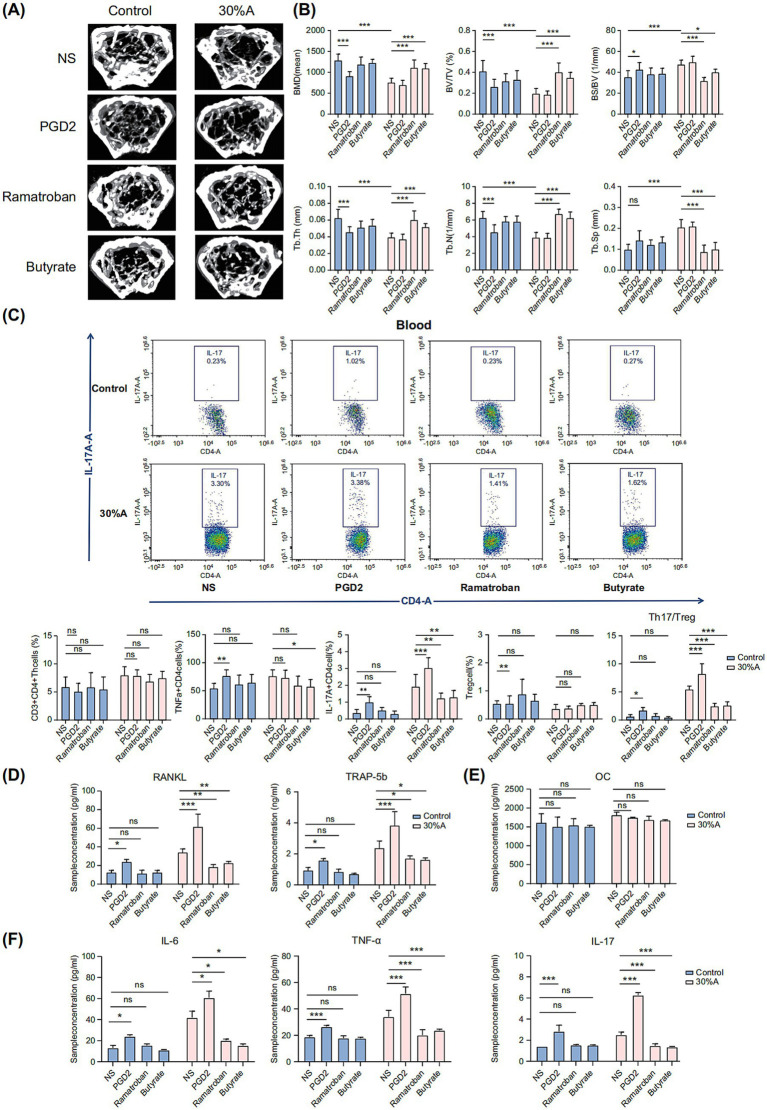
PGD2 induces greater bone loss and elevations in the Th17/Treg ratio in the blood. **(A)** Micro-CT images after the application of PGD2 and ramatroban. **(B)** Bone parameter analysis: BMD, BV/TV, BS/BV, Tb.Th, Tb.N, and Tb.Sp. **(C)** Flow cytometry detection of Th17 cells in the blood and differential blood flow cytometry analysis. **(D)** Osteoclast markers RANKL and TRAP-5b, **(E)** Osteoblast marker OC. **(F)** Inflammatory factors IL-6, TNF-α, and IL-17. Values are represented as bar graphs with means ± SDs. *p < 0.1, **p < 0.05, ***p < 0.01 (*n* = 6). 30% A, 30% alcohol; BMD, bone mineral density; BS/BV, bone surface/bone volume; BV/TV, trabecular volume/tissue volume; CT, computed tomography; IL, interleukin; NS, normal saline; OC, osteocalcin; PGD2, prostaglandin D2; RANKL, receptor activator of nuclear factor kappa beta ligand; SD, standard deviation; Tb.N, trabecular number; Tb.Sp, trabecular separation; Tb.Th, trabecular thickness; TNF, tumor necrosis factor; TRAP, tartrate-resistant acid phosphatase; ns, not significant.

Elevated PGD2 levels may be related to the bone loss observed in AOP mice. To further explore this, we conducted a comparative study using PGD2, its specific inhibitor ramatroban (Ogletree et al., 2022), and butyrate supplementation. PGD2 administration significantly reduced BMD, BV/TV, Tb.Th, and Tb.N (*p* < 0.01) in the control group, with no significant effects in the alcohol-exposed group. Ramatroban treatment significantly increased BMD, BV/TV, Tb.Th, and Tb.N (*p* < 0.01), and significantly decreased BS/BV and Tb.Sp (*p* < 0.01) compared to the saline-treated group. Butyrate supplementation after alcohol consumption had similar effects, significantly improving bone parameters, whereas butyrate supplementation alone did not show a noticeable bone-protective effect (Figures 7A,B).

PGD2 significantly increased the proportion of IL-17(+)CD4(+) T cells and the Th17/Treg ratio in the blood of the 30%-AOP group (*p* < 0.01). In contrast, ramatroban and butyrate treatments significantly decreased IL-17(+)CD4(+) T cells levels (*p* < 0.05) and the Th17/Treg ratio (*p* < 0.01; Figure 7C). Serum analysis revealed that PGD2 supplementation significantly elevated the RANKL and TRAP-5b levels (*p* < 0.01; Figure 7D), while levels of the osteoblast marker OC remained unchanged (Figure 7E). Additionally, PGD2 significantly increased the TNF-α, and IL-17 levels, while ramatroban and butyrate significantly inhibited these inflammatory factors in the 30%-AOP group (Figure 7F). These findings highlight the critical role of PGD2 in alcohol-induced bone loss and confirm the efficacy of the PGD2-targeted inhibitor and the anti-inflammatory agent butyrate in AOP.

## 4 Discussion

This study systematically reveals the core mechanisms of AOP from the perspectives of immune regulation and metabolism. It also analyzes the spatial distribution and functional differences of normal gut microbiota, elucidating how alcohol triggers metabolic disturbances in the intestinal microenvironment, systemic immune imbalance, and gut microbiota dysbiosis. The results demonstrate that alcohol significantly activates osteoclasts, enhancing bone resorption while having minimal impact on osteoblast function. This suggests that the marked elevation of PGD2 is the primary pathological feature driving abnormal bone resorption.

Using 16S rRNA sequencing and metabolomic techniques, this study provides a detailed analysis of gut microbiota distribution and metabolic characteristics across different intestinal segments in normal and alcohol-treated mice, highlighting significant differences between the upper and lower GI tracts. In normal mice, the gut microbiota displays high diversity and complexity, with dynamic interactions among microbial species serving as the foundation for host immune homeostasis and metabolic health. This spatially specific distribution of gut microbiota underpins the functional differences across intestinal segments. Significant differences in microbial populations across various intestinal regions and feces, suggesting that relying solely on the microbiota from the colon or feces as representatives of the overall gut microbiota structure is insufficiently accurate. Although microbial abundance is higher in the lower digestive tract, its diversity is markedly lower than that in the upper digestive tract, particularly in the duodenum, which exhibits significantly greater diversity.

The upper GI tract is characterized by higher microbial diversity and a large number of functional expressions, supporting key physiological functions such as local immune regulation and nutrient absorption. This region’s sensitivity to external disturbances makes it a potential target for precision therapy, although it also poses challenges for research accuracy. Using colonic or fecal microbiota as representatives of the entire intestinal microbiome may overlook critical information about the upper GI tract’s unique core microbiota. Conversely, the lower GI tract features lower microbial diversity but higher microbial abundance and metabolic product accumulation. Microbial populations in the lower GI tract primarily support fermentation, producing metabolites such as short-chain fatty acids (SCFAs) that reflect overall intestinal metabolic activity (Van der Hee and Wells, 2021). However, the lower gut is more resilient to disturbances, with its microbiota exhibiting higher adaptability. These differences arise from the distinct anatomical structures and microenvironments of the upper and lower GI tracts, such as variations in oxygen levels, pH, and fermentation conditions. The cooperative division of metabolic and immune functions across these regions ensures the maintenance of intestinal homeostasis.

In the context of the observed intestinal mucosal damage and increased permeability in the AOP model (Livshits and Kalinkovich, 2022), we speculate that alcohol may induce bone loss through the gut-bone axis. This may occur by disturbances to immune cells in the submucosa, and based on alcohol-induced gut microbiota dysbiosis and metabolic disturbances that exacerbate the functional imbalance across intestinal segments, via both metabolic and immune pathways.

Alcohol intake significantly altered the microbiota structure in different intestinal regions and feces of mice. At the phylum level, the abundance of microbiota in different intestinal segments showed both increases and decreases. Taking the duodenum as an example, the abundances of *Campylobacterota* and Patescibacteria decreased, while that of *Actinobacteriota* increased. Overall, alcohol had a relatively limited impact on the microbial composition at the phylum level in different intestinal segments, indicating that the overall intestinal microbial community has a certain degree of anti-interference ability and stability (Adak and Khan, 2019). Even after the impact and screening of alcohol on the microbiota in each intestinal segment, the change in microbiota structure was not as drastic as expected. However, at the genus and species levels, the number of microbiota differences within different intestinal segments was extremely considerable. The cumulative effects of these subtle differences may have a remarkably significant impact on the intestinal microenvironment.

When examining the microbiota at the genus and species levels, there are substantial differences among different intestinal segments. The cumulative impact of these subtle disparities on the intestinal microenvironment can be profound. At the genus level, an analysis of the common flora in each region reveals that alcohol led to a significant reduction in the abundance of *Candida*, *Saccharomyces*, and *Lactobacillus* in the duodenum, jejunum, and ileum. Conversely, the levels of *Dubosiella* and *Bifidobacterium* increased notably in these same segments. These changes suggest that although alcohol affects the gut microbiota at specific sites in a targeted manner, intricate interconnections exist among these different locations. Overall, the key difference lies in the fact that alcohol diminishes beneficial genera in the upper digestive tract while promoting the growth of pathogenic genera in the lower digestive tract. The functional prediction analysis of the alcohol-disrupted flora, utilizing metabolomics and the KEGG, indicates that functions related to arachidonic acid, such as the activity of PGD2, are heightened, while the function of short-chain fatty acid secretion, exemplified by butyrate, is weakened in the disrupted flora. As a result, the intestinal environment disturbed by alcohol evolves into an inflammatory state (Patel et al., 2015).

The intestinal barrier serves as a crucial defense, separating the intestinal contents, which may contain numerous contaminants, from the flora within the intestine. If the intestinal contents or metabolites penetrate the cells of the lower intestinal wall or mesenteric lymph nodes (Peyer’s patches), they may trigger the activation of the immune pathway associated with AOP. Studies on the damage caused by alcohol to the intestinal wall have observed a significant increase in intestinal length. These findings suggest that alcohol has a notable impact on the extensibility of the intestine in mice, potentially by disrupting the tight junction proteins in the intestinal wall, leading to increased intestinal extensibility and cell loosening. Hematoxylin and eosin staining sections of different intestinal segments reveal that the degree of injury to the intestinal walls of the upper digestive tract, including the duodenum and jejunum, is more severe than that of the colonic wall in the lower digestive tract. This disparity may be attributed to the fact that alcohol absorption in the intestinal tract primarily occurs in the esophageal mucosa, stomach, duodenum, and jejunum (the upper digestive tract). These results further confirm that the effect of alcohol on the intestinal tract of mice is region-specific. It has been observed that alcohol causes the cells between the intestinal walls to thin and the gaps between them to widen, directly demonstrating that alcohol disrupts the structural integrity of the intestinal mucosa. Once alcohol impairs the barrier function of the intestinal tract, it is inevitable that there will be potential subsequent impacts on the digestive, absorptive, and immune regulatory functions of the intestine. The elevation of DAO and LPS levels provides additional evidence that alcohol damages the integrity of the intestinal barrier. This compromise makes it easier for bacteria and other components to cross the intestinal barrier and enter the intestinal wall, subsequently being transported to other parts of the body via the bloodstream. As an endotoxin, LPS has the potential to activate the immune system, triggering either systemic or local inflammatory responses (Candelli et al., 2021).

Alcohol disrupts the microbiota environment, contributing to AOP in multiple ways. It damages the microbiota, weakening their protective and reparative functions for the intestinal barrier. On the other hand, it promotes the secretion of more toxic substances, such as LPS, by harmful flora while inhibiting beneficial bacteria from producing anti-inflammatory antioxidants, such as short-chain fatty acids. This leads to the accumulation of inflammatory factors in the intestine, which then penetrate the intestinal wall and activate the immune system, either systemically or locally, including in the bones, ultimately leading to the development of AOP.

The decline in upper GI microbial diversity weakens local immune regulation, leading to excessive production of pro-inflammatory factors like PGD2, IL-17, and TNF-*α*. These factors stimulate osteoclast activity, enhancing bone resorption and accelerating BMD loss. Furthermore, lower gut dysbiosis, including the reduction of SCFA-producing bacteria and key metabolites like acetate and butyrate, disrupts systemic metabolic balance and bone metabolism. SCFAs play a critical role in modulating Treg cell balance, suppressing inflammation, and regulating the gut-bone axis. Their depletion amplifies the Th17/Treg imbalance, further driving immune dysregulation and AOP progression.

The segment-specific nature of alcohol-induced dysbiosis suggests that interventions for AOP should focus on targeted regulation. Restoring upper GI microbial balance may benefit from localized anti-inflammatory treatments or microbiota transplantation, while improving lower gut function could involve probiotics or SCFA precursors to enhance metabolic activity. Combining these approaches with PGD2 inhibitors may provide multi-target therapeutic strategies addressing both metabolic and immune pathways.

After supplementation with *R. intestinalis*, there was a significant supportive effect on the bone density of AOP in mice. In contrast, the short-term alcohol abstinence model did not significantly promote bone mass recovery. This result indicates that *R. intestinalis* is a promising probiotic treatment regimen for AOP. However, it should be noted that the *R. intestinalis* treatment experiment was conducted after the mice had abstained from alcohol, and the therapeutic effect on mice that are still drinking alcohol has not been verified. Since it cannot be ruled out that alcohol may inactivate or inhibit *R. intestinalis*, thereby reducing its efficacy, the treatment of AOP with *R. intestinalis* may only be applicable to those who have abstained from alcohol and is difficult to serve as a prevention for osteoporosis in the general population of heavy drinkers.

However, this study has some limitations. Firstly, the research results are based on a mouse model. Although mouse models are widely used and of great value in medical research, there are inherent differences between mice and humans in physiological structures, metabolic mechanisms, and the composition of the microbial community. Secondly, the sampling frequency was relatively low, and samples were only collected at specific time points. The impact of alcohol on the gut microbiota may be a gradual and long-term process and prolonged drinking can reveal more of these complex adaptive changes, which may have a profound impact on the development of AOP. Due to the lack of long-term data, we were unable to determine whether the gut microbiota dysbiosis and related physiological changes observed in this study are persistent and their mechanisms of action in the chronic progression of the disease. Thirdly, 16S rRNA sequencing can only reflect the taxonomic information of bacteria and has limited ability to detect other microorganisms such as fungi and viruses. Ignoring the changes of other microorganisms may lead to an incomplete assessment of the overall gut microbiota dysbiosis. Due to the limited sensitivity and coverage of detection techniques in metabolomic analysis, some low-abundance metabolites may not be detected. These low-abundance metabolites may play an important role in intestinal metabolism and signaling pathways related to bone health. Fourthly, in the exploration of the mechanism of Th17 cells in the downstream immune pathway, this study did not use Th17 gene-knockout (Th17−/−) mice for verification, making the elaboration of this mechanism lack more direct and powerful evidence support. The long-term safety and potential side effects of ramatroban, butyrate, *and R. intestinalis* involved in the experiment have not been fully evaluated.

Future exploration of the potential therapeutic effects of probiotics on AOP is necessary. Using metagenomics and bioinformatics, high-throughput screening can be carried out to accurately select strains from the microbial resources of diverse ecological environments that can used to effectively resist alcohol interference and regulate the gut microecology. Similarly, CRISPR-Cas technology can be used to genetically edit existing strains to enhance their ability to produce SCFAs or improve their adhesion to intestinal epithelial cells (Zetsche et al., 2015) and develop composite formulas containing multiple strains and prebiotics. Through the complementary functions of different strains and the promotion of growth and reproduction of probiotics, a multi-target synergistic treatment for AOP can be achieved.

In clinical practice, many female patients with osteoporosis have a history of long-term alcohol consumption; however, our experiment involved only male mice in the research model for AOP. Bone metabolism in female mice is highly regulated by estrogen, which is affected by alcohol consumption (Zallar et al., 2024). Estrogen can effectively inhibit the activity of osteoclasts and promote the function of osteoblasts, playing a core role in maintaining bone mass balance. The impact of alcohol on estrogen levels and the interaction between estrogen level fluctuations and the gut microbiota-immune-bone axis may be quite different in female compared to male mice (Zheng et al., 2023). Using only male mice in this experiment makes it impossible to explore these complex mechanisms related to estrogen, resulting in a one-sided understanding of the pathogenesis of AOP. The research results are difficult to comprehensively demonstrate the changes in bone metabolism of different-sex individuals under the action of alcohol. Additionally, research indicates that the microbial diversity in the intestines of female mice may be higher than that of male mice, and the abundances of some specific microbial groups also vary by sex (Peters et al., 2022). These differences may lead to differences in the tolerance, metabolic patterns of the gut microbiota in female mice to alcohol, and their interaction patterns with the host immune system compared to male mice. Since female mice were not included in this experiment, it is impossible to clarify the role of these sex-specific gut microbiota characteristics in the development of alcoholic osteoporosis.

In the future, we will construct a sex-mixed animal model, that is, establish an experimental system that includes both female and male mice. Using comparative analysis we intend to set up groups of different alcohol exposure doses and time gradients, and closely observe the differences in metabolism and outcomes in female and male mice. This will help clarify the sex-differences in AOP, providing solid basic data for in-depth understanding of the pathogenesis. By detecting the expression changes in sex-hormone-related genes (such as estrogen receptor genes, androgen receptor genes, etc.) (Wu et al., 2017), we will deeply analyze the differences in the regulation of these gene expressions among sex, reveal sex-specific molecular mechanisms in the pathogenesis of AOP, and provide a strong theoretical basis for the screening of targeted treatment targets.

Given that current experiments all rely on animal models, rigorous clinical studies should be carried out before applying our findings. Specifically, multi-center, large-sample randomized, double-blind, controlled trials, involving patients with AOP, should be carried out to comprehensively evaluate the effectiveness and safety of probiotic and PGD2 inhibition therapy. Similarly, long-term follow-up monitoring patients’ skeletal health status (including bone density and bone microstructure), gut microbiota, and metabolites is important. This data will help formulate targeted and adaptable treatment plans. In addition, personalized treatment plans, according to the patients’ gut microbiota, genetic polymorphisms, and alcohol metabolism conditions, should be developed. Precision medicine should be used to improve the treatment effect of AOP and reduce side effects. The synergistic effects of probiotics/anti-PGD2 drugs and traditional anti-osteoporosis drugs should be explored, and the combined medication plan should be optimized to improve patient outcomes. Lifestyle intervention measures such as alcohol abstinence, nutritional improvement, and exercise should be combined to improve the gut microbiota, bone metabolism, and overall health status of patients with AOP.

Finally, the possibility of combining probiotics, anti-PGD2 drugs with emerging therapies (such as gene therapy and stem cell therapy) should be explored. Through this interdisciplinary approach, new treatment avenues for AOP can be found, benefiting patients and promoting the development of this field.

In conclusion, this study reveals that the gut and fecal microbiota of healthy mice exhibit spatial complexity and local consistency, suggesting that relying solely on fecal or colonic microbiota and metabolites to represent the overall gut environment is inappropriate. Alcohol consumption induces distinct disruptions in the microbiota and metabolites across different gut regions and fecal matter, yet certain metabolic changes, such as increased PGD2 production, tend to converge across gut segments. Elevated PGD2 levels stimulate Th17 cells and promote osteoclastogenesis, exacerbating bone loss. Supplementation with *Roseburia intestinalis* and inhibition of PGD2 effectively mitigate bone deterioration, confirming the critical role of the PGD2–IL-17–osteoclast pathway. These findings highlight the potential of targeting gut microbiota and its metabolic pathways as a therapeutic strategy for alcohol-induced bone disorders.

## Data Availability

The datasets related to 16S rRNA and metabolomics generated for this study can be found in Metabolight database, accession number MTBLS11887, https://www.ebi.ac.uk/metabolights/editor/MTBLS11887/descriptors, NCBI database accession numbers PRJNA1195704 and PRJNA1196152, https://www.ncbi.nlm.nih.gov/bioproject/PRJNA1195704/; https://www.ncbi.nlm.nih.gov/bioproject/?term=PRJNA1196152.

## Footnotes

^1^ https://www.genome.jp/kegg/pathway.html

## Glossary

ALB: albumin
ANOSIM: analysis of similarities
AOP: alcoholic osteopenia
BMD: bone mineral density
BS/BV: bone surface/bone volume
BV/TV: bone volume/tissue volume
DAO: diamine oxidase
GI: gastrointestinal
IL: interleukin
LPS: lipopolysaccharide
micro-CT: micro-computed tomography
MRPP: multi-response permutation procedure
NMDS: Non-Metric Multidimensional Scaling
OC: osteocalcin
OUT: operational taxonomic unit
PCoA: principal coordinates analysis
P1NP: procollagen type I N-terminal pro-peptide
PGD2: prostaglandin D2
RANKL: receptor activator of nuclear factor kappa beta ligand
SCFAs: short-chain fatty acids
SD: standard deviation
Tb.N: trabecular number
Tb.Sp: trabecular separation
Tb.Th: trabecular thickness
TNF: tumor necrosis factor
TRAP: tartrate-resistant acid phosphatase
