# Supplementary material for: Alcohol-induced bone loss driven by dysregulated spatial distribution of gut microbiota and PGD2-IL17 pathway-mediated osteoclast activation
Source: Front Microbiol. 2025 May 15;16:1551028. doi: 10.3389/fmicb.2025.1551028 (PMC12121995; doi:10.3389/fmicb.2025.1551028)
Supplement: Supplementary file 1 [file Data_Sheet_1.docx]

Supplementary Material

# Supplementary Figures and Tables

## Supplementary Figures


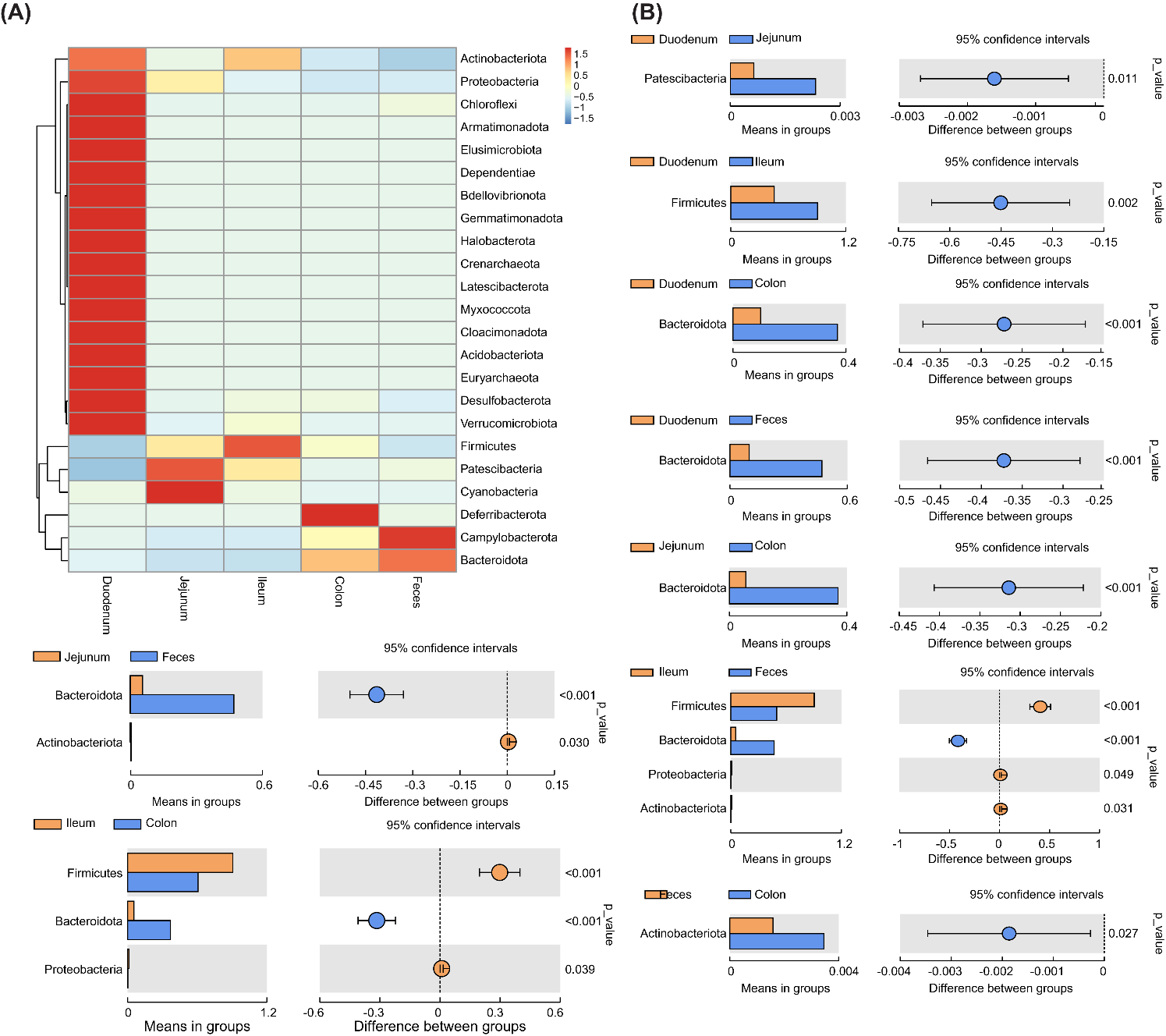


**Supplementary Figure 1.** Differences at the phylum level among different intestinal segments and feces in normal mice. (**A**) Phylum-level species abundance clustering heatmap, (**B**) Phylum-level T-test (Duodenum, Jejunum), (**C**) Phylum-level T-test (Ileum, Colon)

**
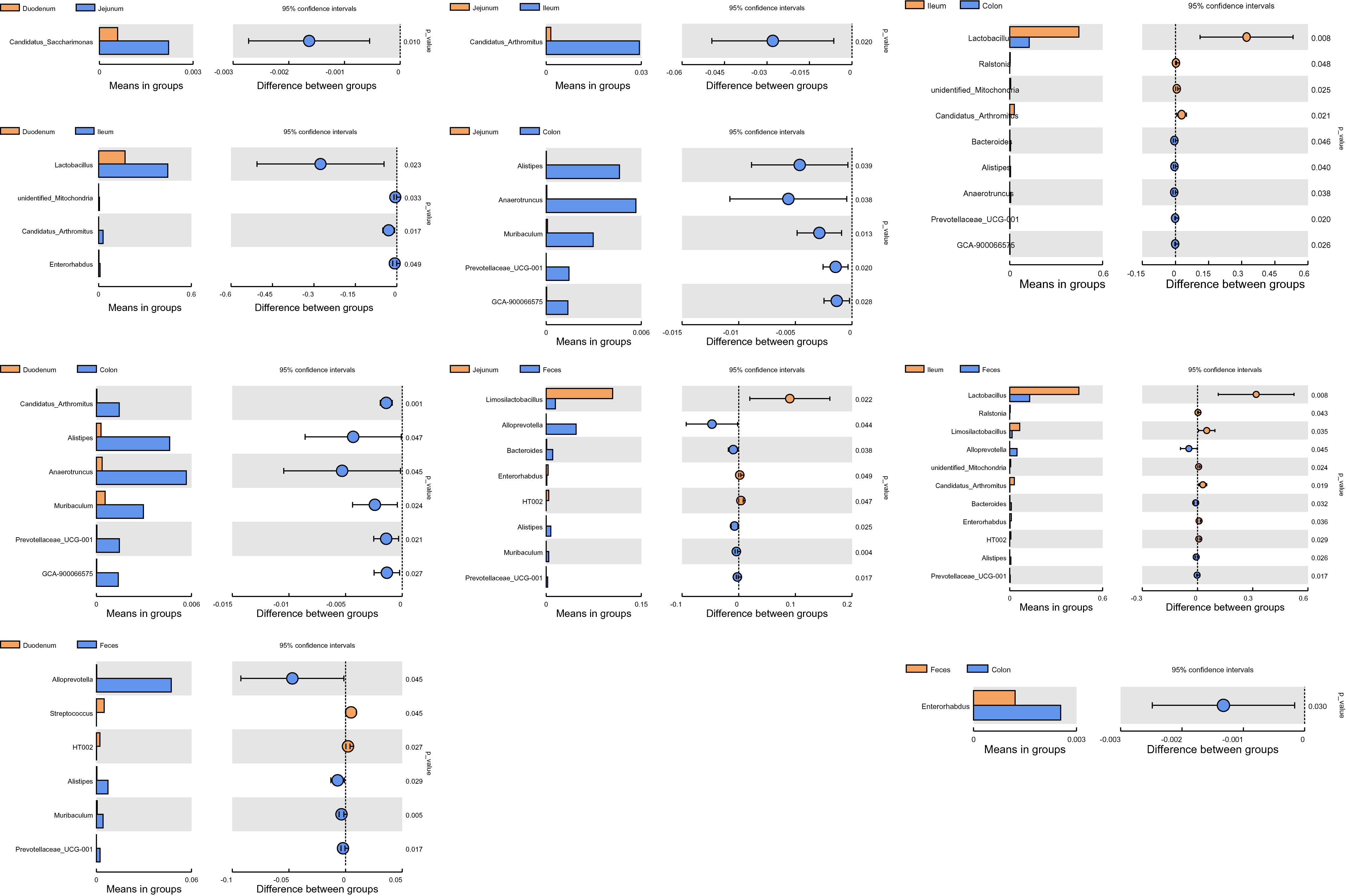
**

**Supplementary Figure 2.** T-test at the genus level among different intestinal segments and feces in normal mice (**A**) T-test (Duodenum) (**B**) T-test (Jejunum) (**C**) T-test (Ileum, Colon)

**
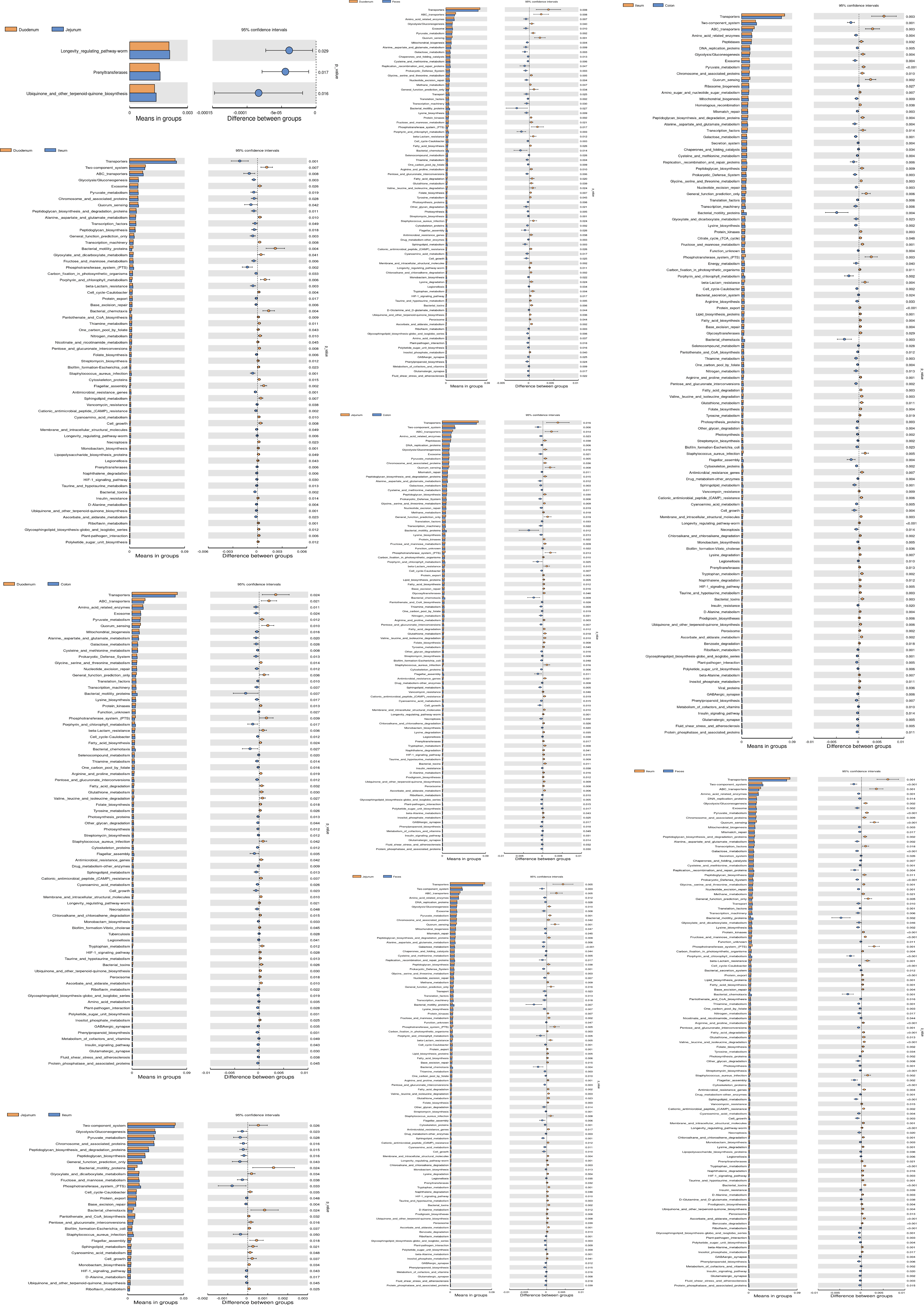
**

**Supplementary Figure 3.** T-test at KEGG level 3 (p < 0.01) among different intestinal segments and feces in normal mice (**A**) T-test (Duodenum) (**B**) T-test (Jejunum) (**C**) T-test (Ileum)





**Supplementary Figure 4.** T-test at KEGG level k (p < 0.01) among different intestinal segments and feces in normal mice. (**A**) Heatmap of KEGG level k across different intestinal segments and feces, (**B**)T-test (Duodenum) (C) T-test (Jejunum) (D) T-test (Ileum) (E)T-test (Colon)

**
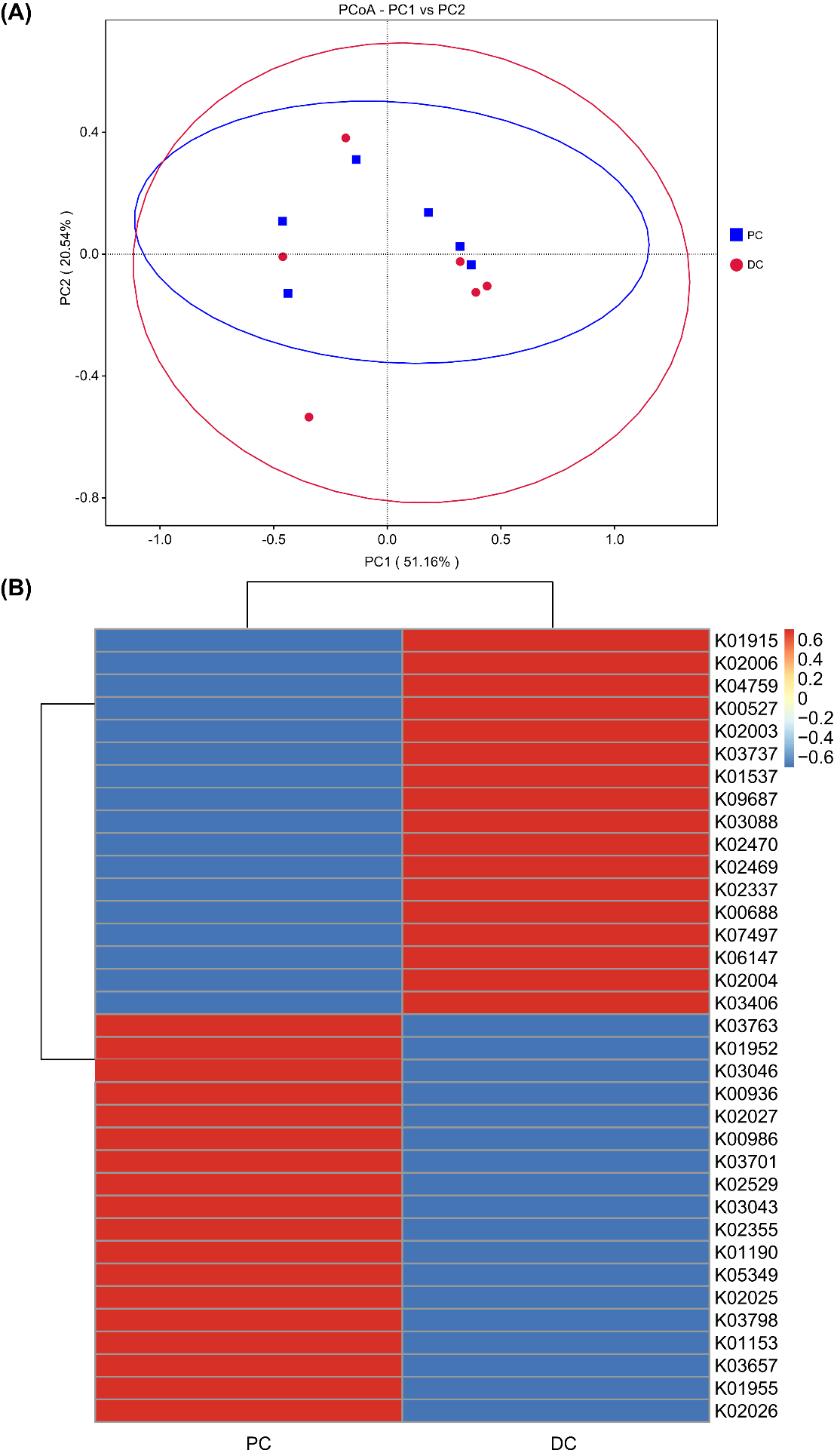
**

**Supplementary Figure 5.** PCOA and KEGG Level K analysis of the proximal and distal colon segments in normal mice (**A**) PCOA of the proximal and distal colon segments in normal mice (**B**) KEGG Level K analysis of the proximal and distal colon segments in normal mice


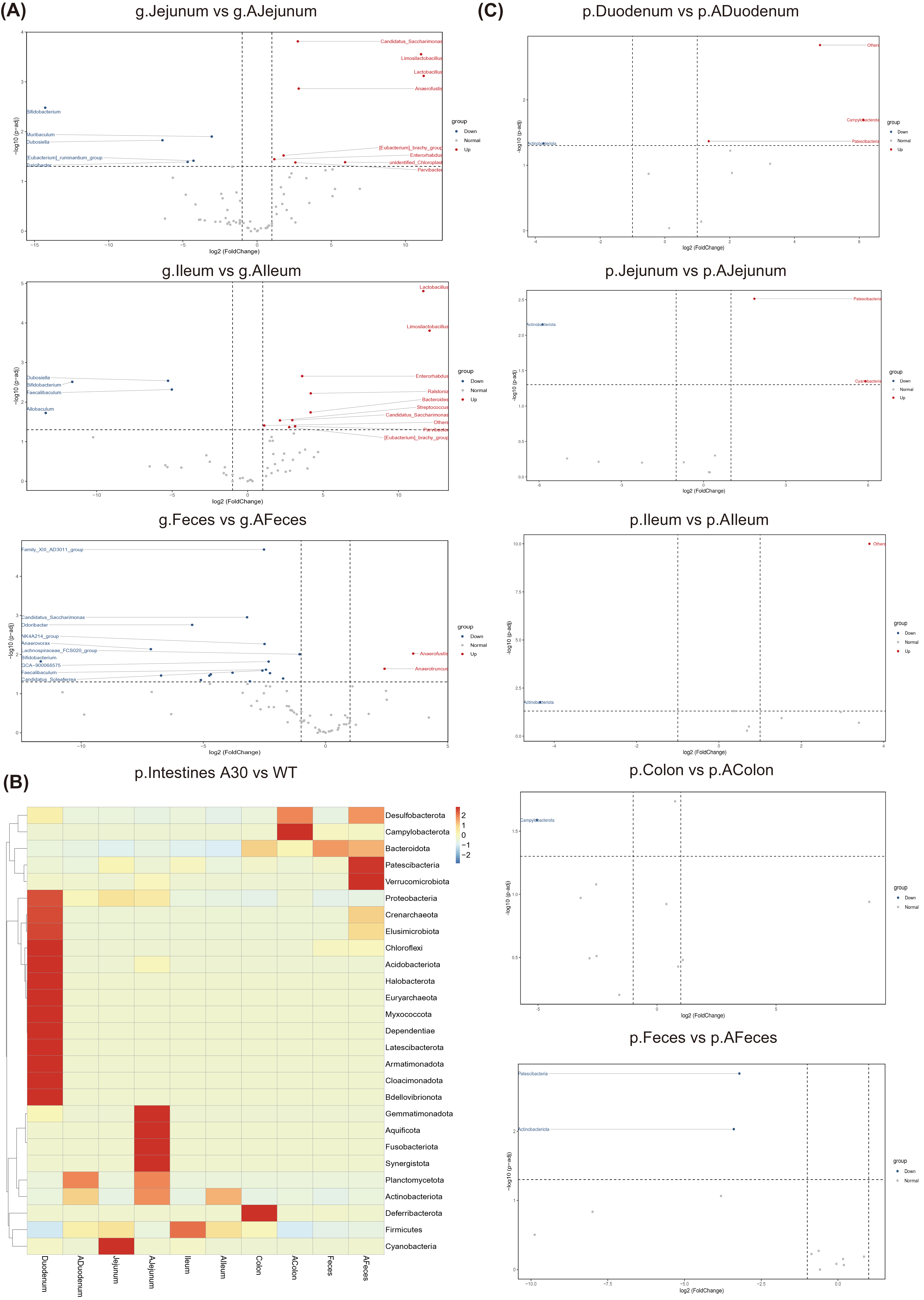


**Supplementary Figure 6.** Differences in the gut microbiota across the entire intestinal tract of alcohol-consuming mice (**A**) Metastat volcano plot at the genus level for alcohol-consuming mice, (**B**) Phylum-level heatmap for alcohol-consuming mice, (**C**) Metastat volcano plot at the phylum level for alcohol-consuming mice


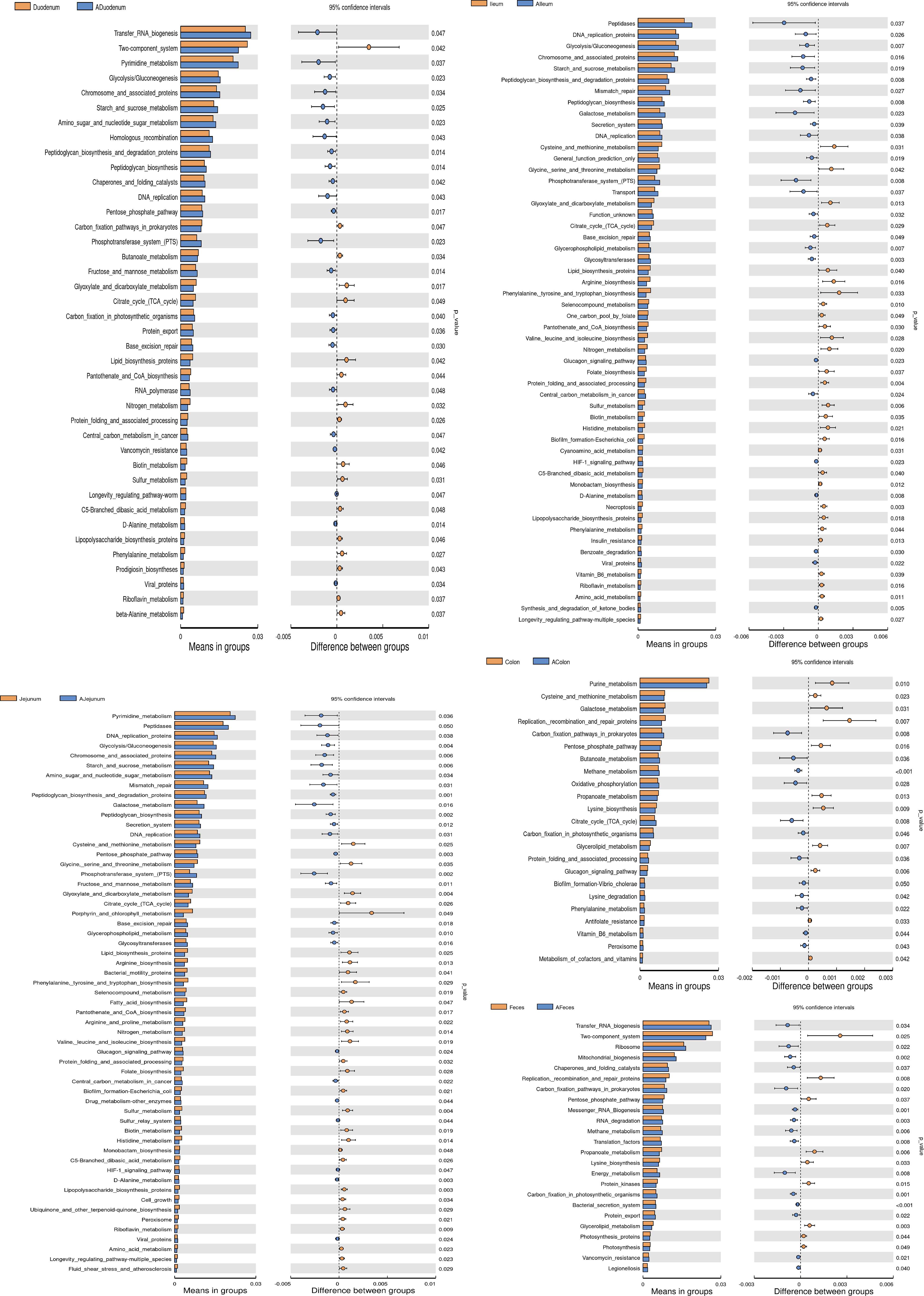


**Supplementary Figure 7.** KEGG level k t-test analysis (p < 0.01) of different intestinal segments in alcohol-consuming mice

**
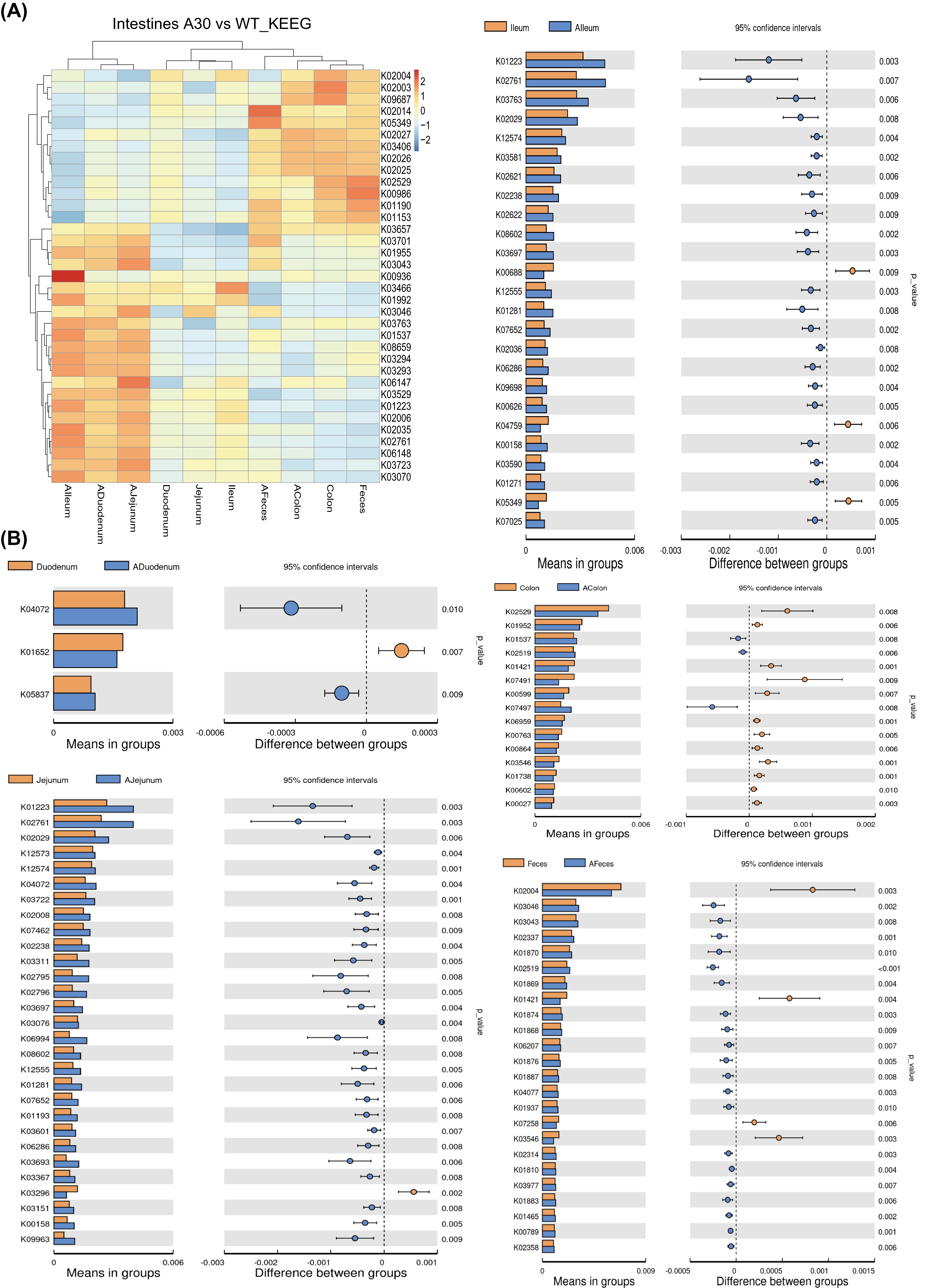
**

**Supplementary Figure 8.** KEGG analysis at Level K for different intestinal segments in alcohol-consuming mice. (**A**) KEGG Level K heatmap, (**B**) KEGG Level K analysis (p < 0.01)


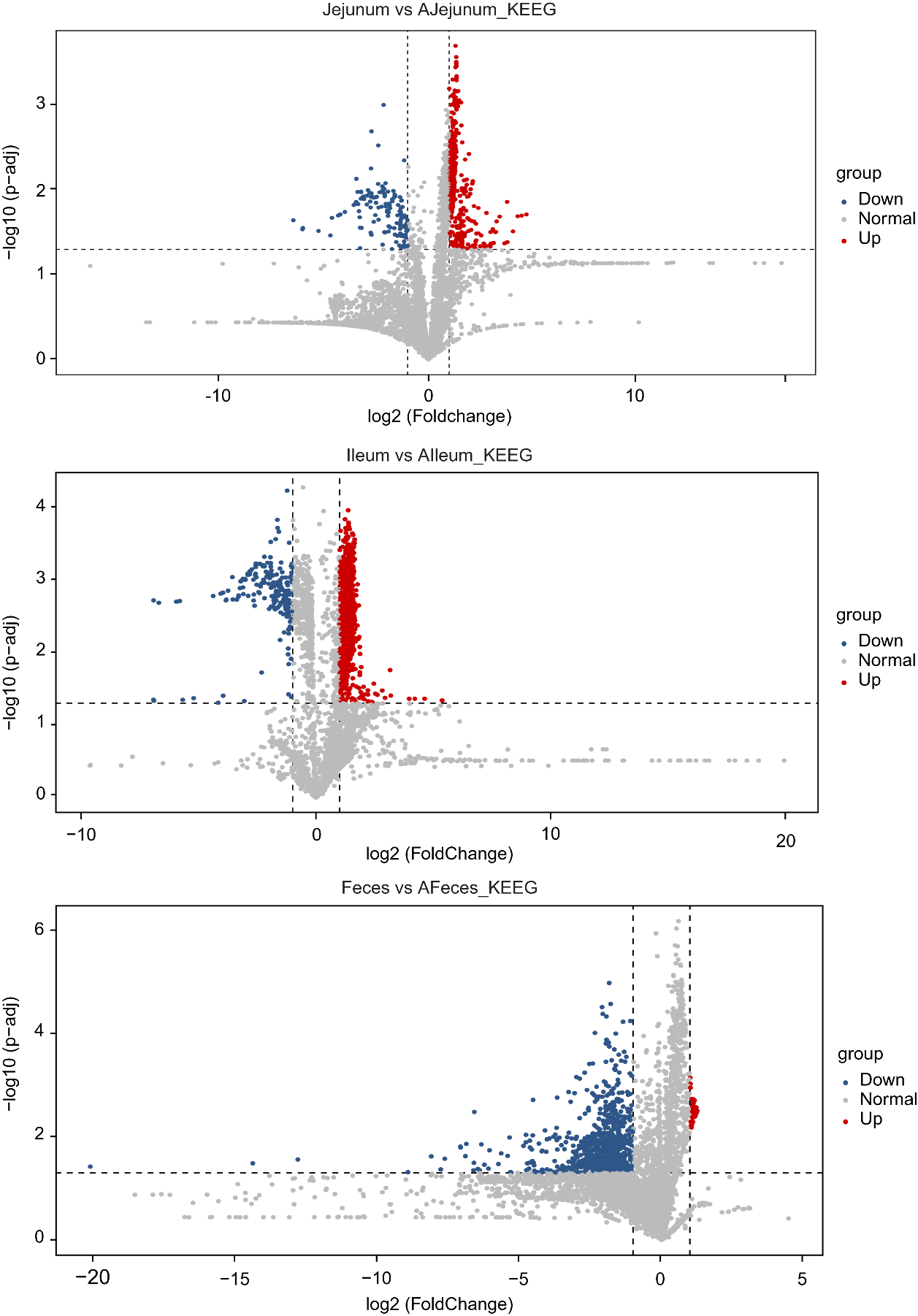


**Supplementary Figure 9.** Functional volcano plot of different intestinal segments and feces in alcohol-consuming mice

**
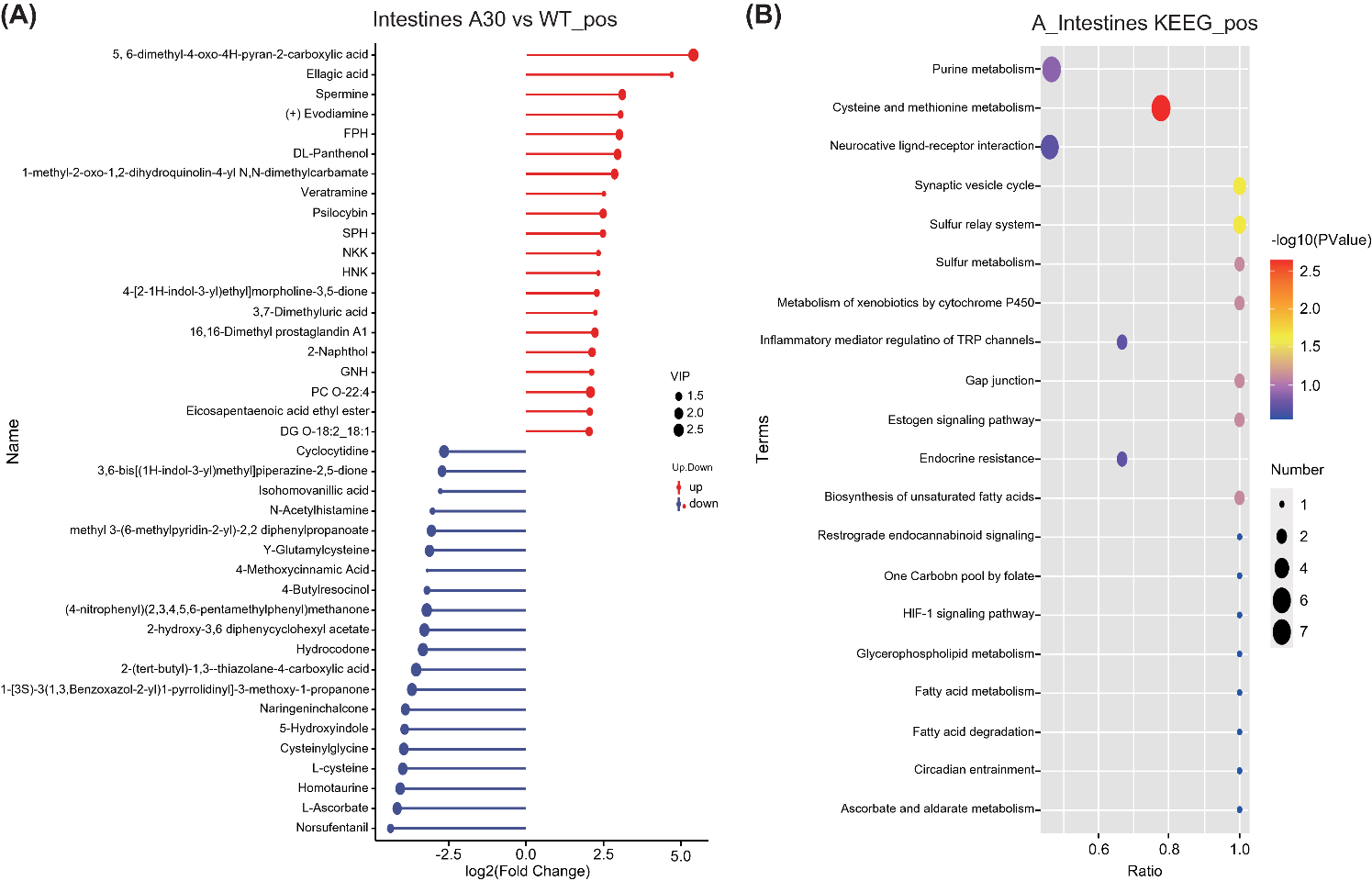
**

**Supplementary Figure 10.** Positive (POS) metabolite matchstick diagram and KEGG metabolic map for the entire intestinal tract of alcohol-consuming mice. (**A**) POS metabolite matchstick diagram for the entire intestinal tract of alcohol-consuming mice, (**B**) KEGG map of POS metabolites in alcohol-consuming mice


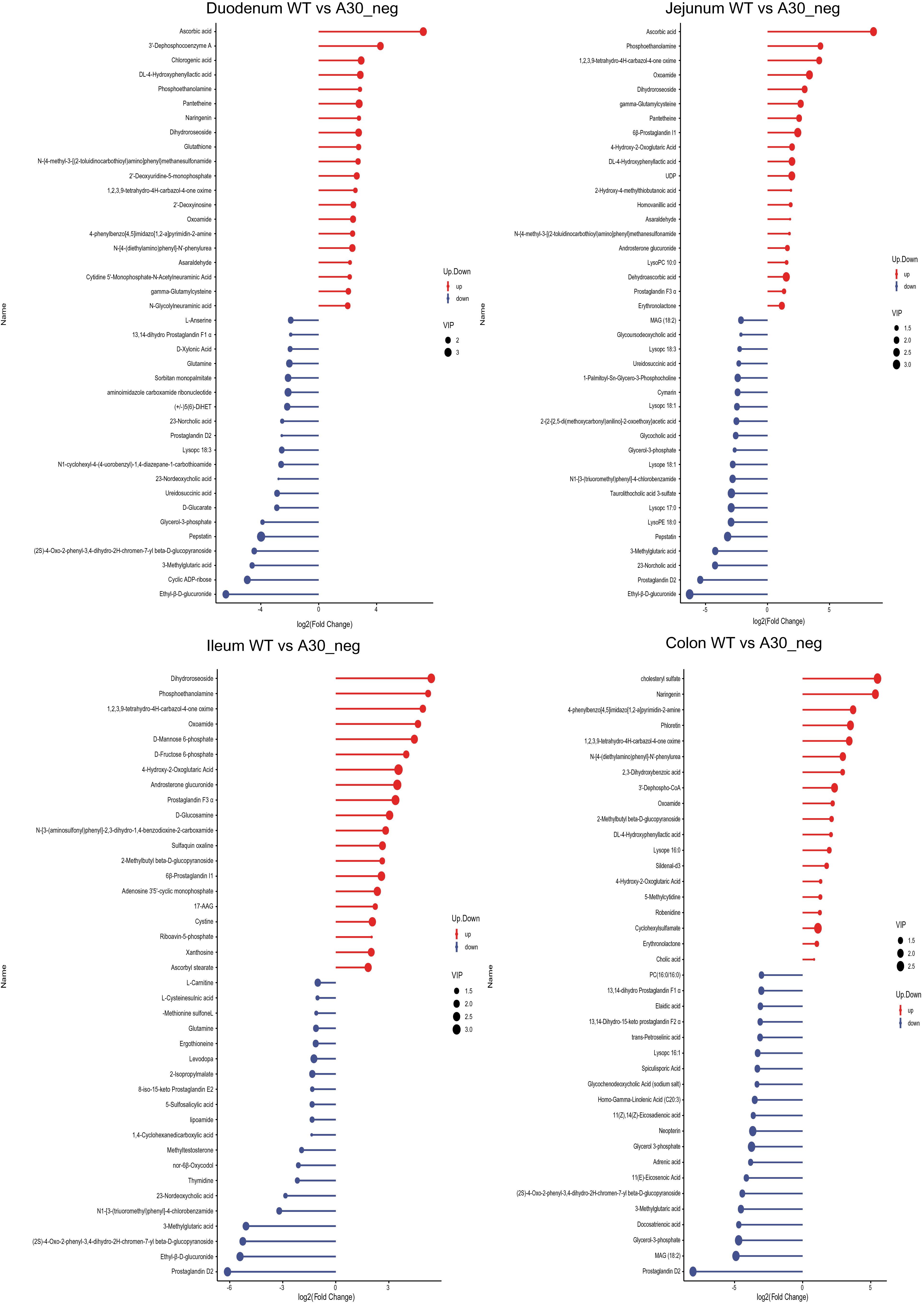


**Supplementary Figure 11.** NEG matchstick diagram for different intestinal segments of alcohol-consuming mice (WT vs 30%-AOP)

**
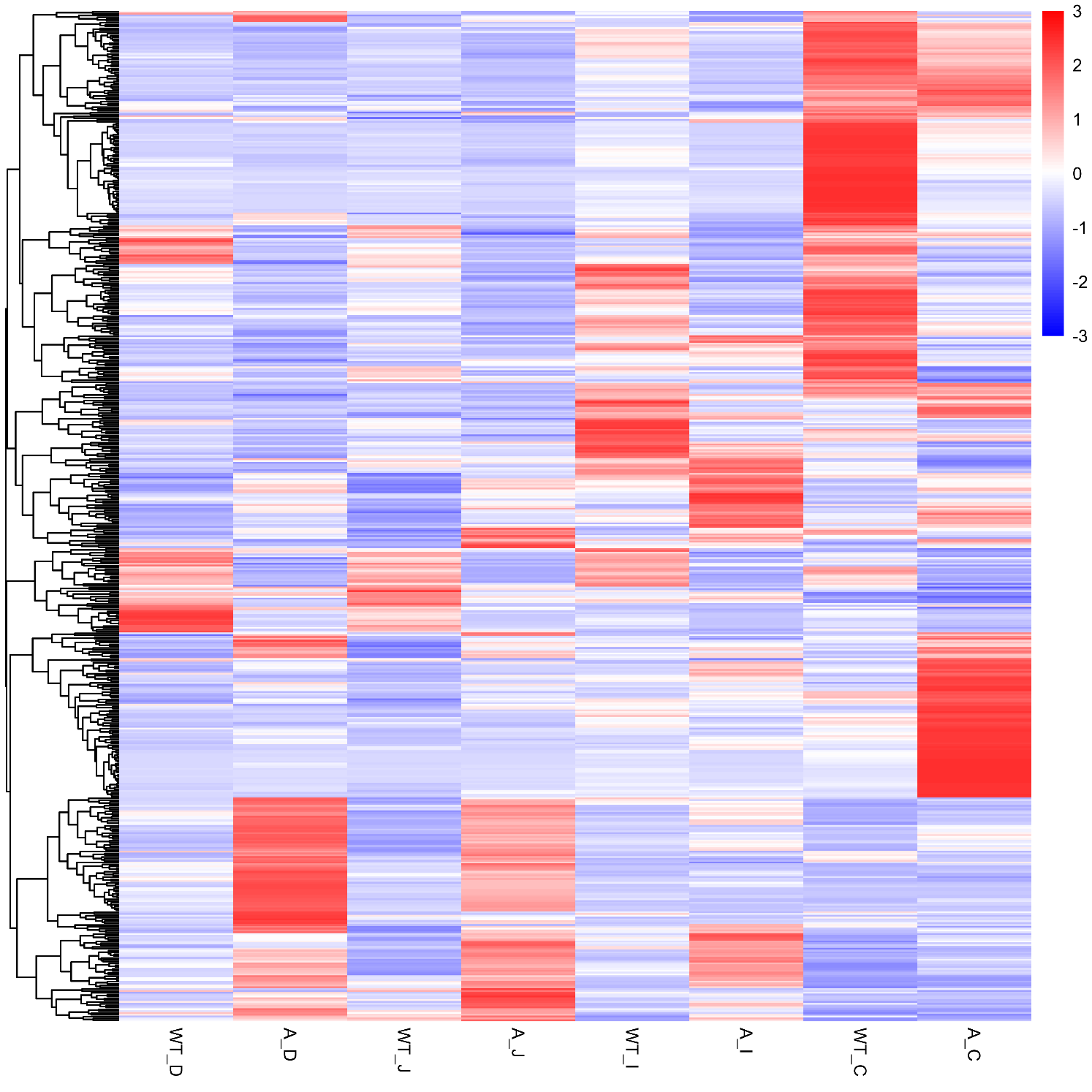
**

**Supplementary Figure 12.** POS heatmap for different intestinal segments of alcohol-consuming mice


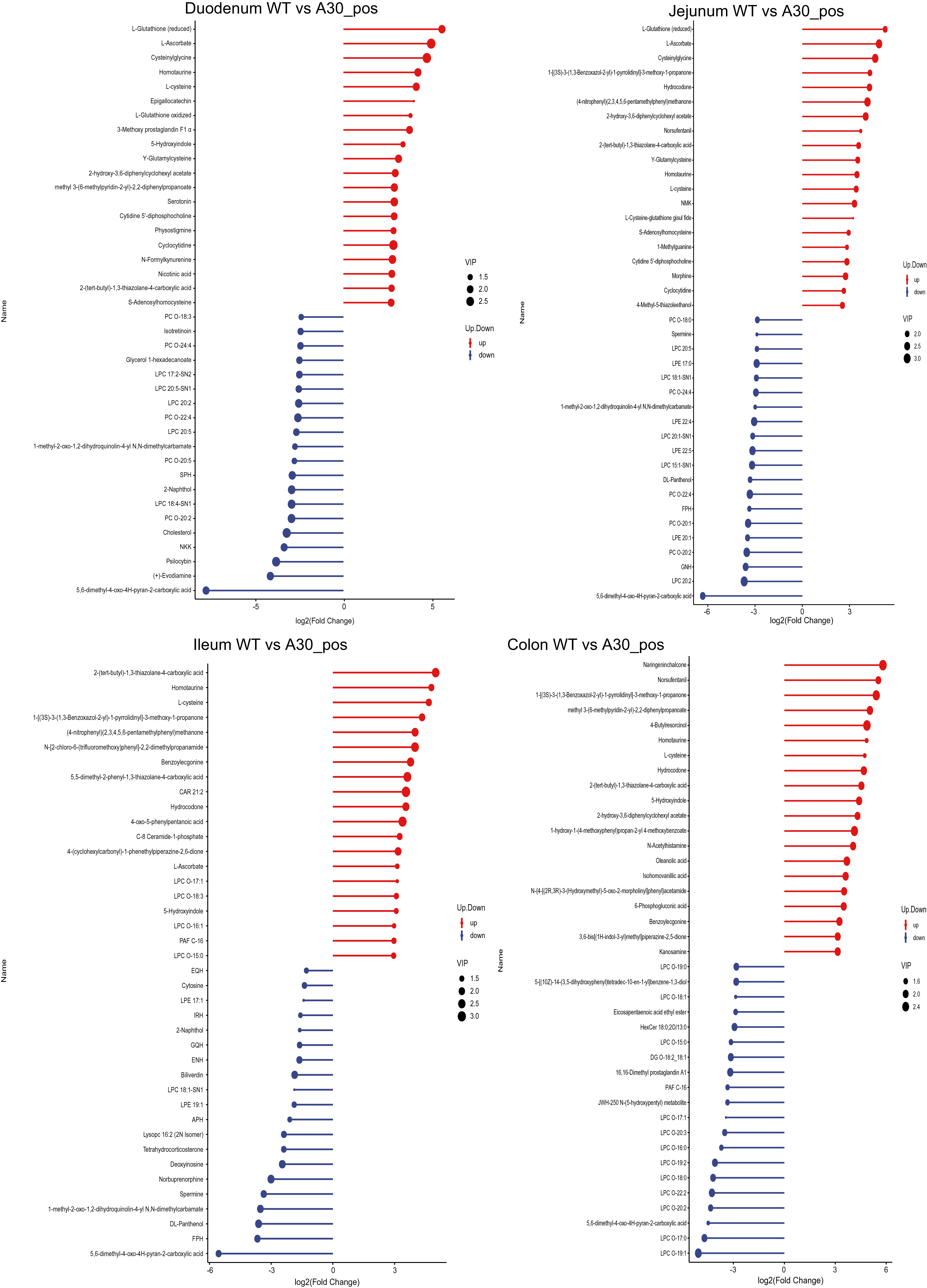


**Supplementary Figure 13.** POS matchstick diagram for different intestinal segments of alcohol-consuming mice (WT vs 30%-AOP)


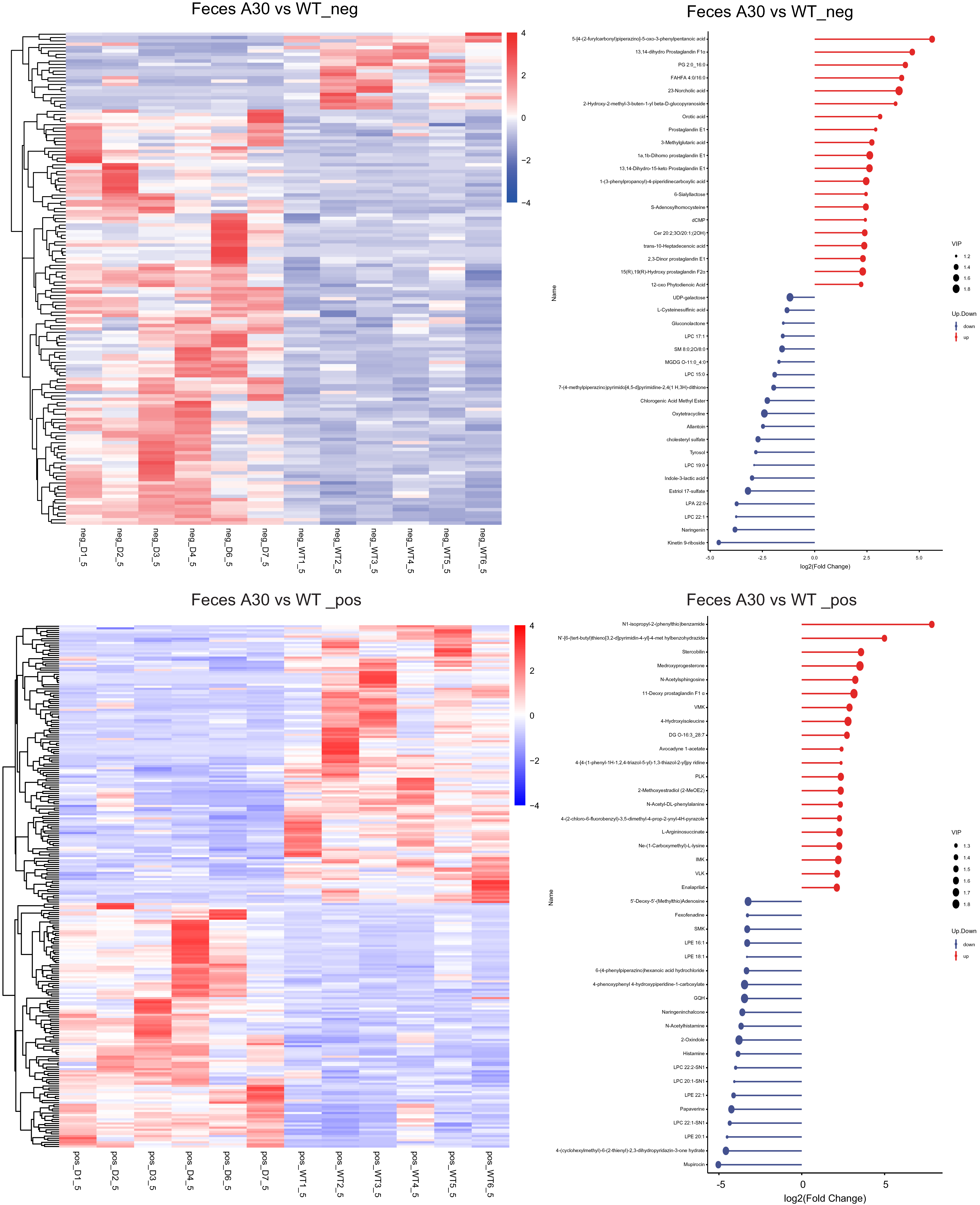


**Supplementary Figure 14.** Differences in fecal metabolites of alcohol-consuming mice (30%-AOP vs WT) (A) Negative (NEG) metabolites (B) positive (POS) metabolites


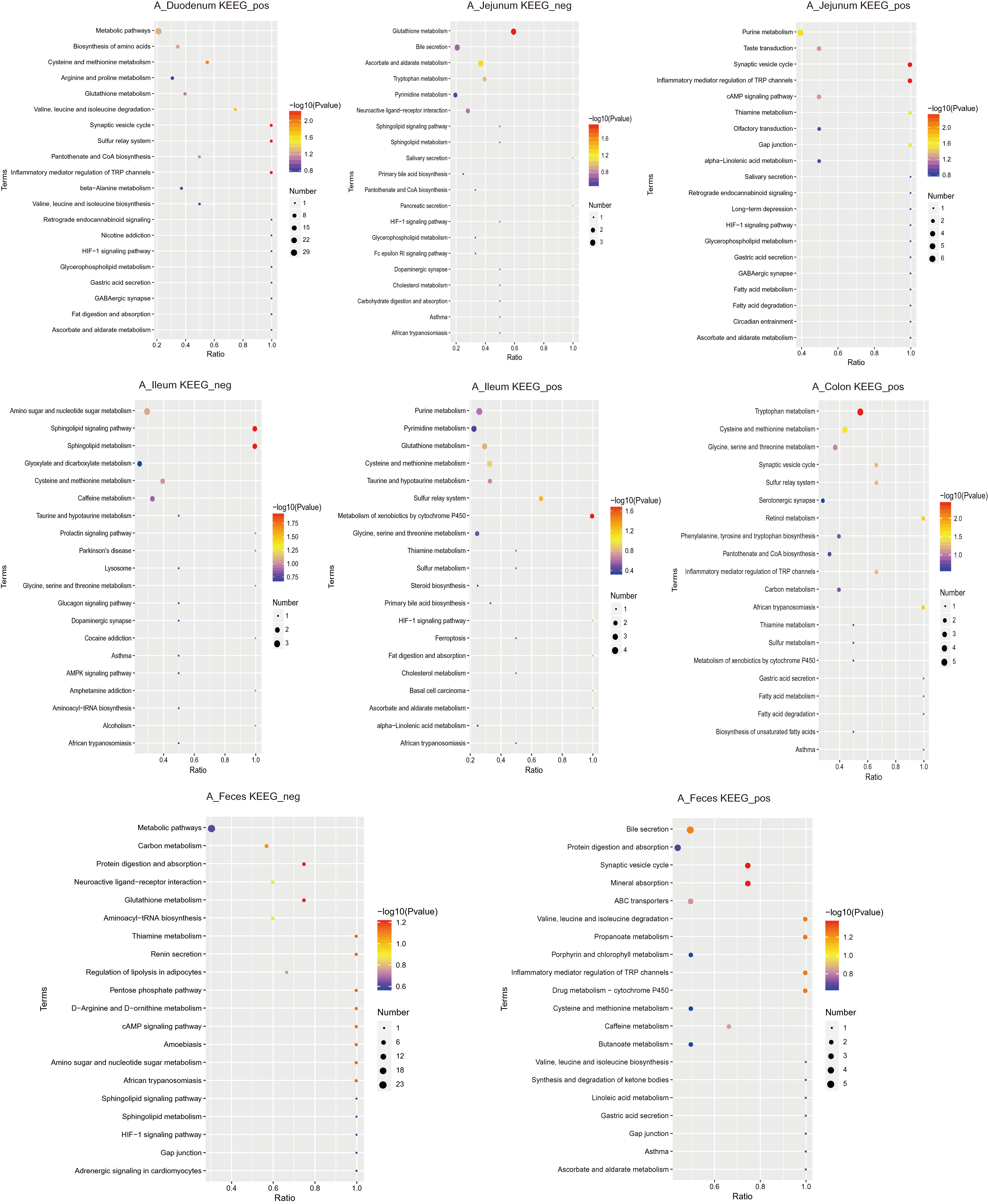


**Supplementary Figure 15.** Functional bubble diagram of different intestinal segments and feces in alcohol-consuming mice (A) Different intestinal segments (B) Feces

**
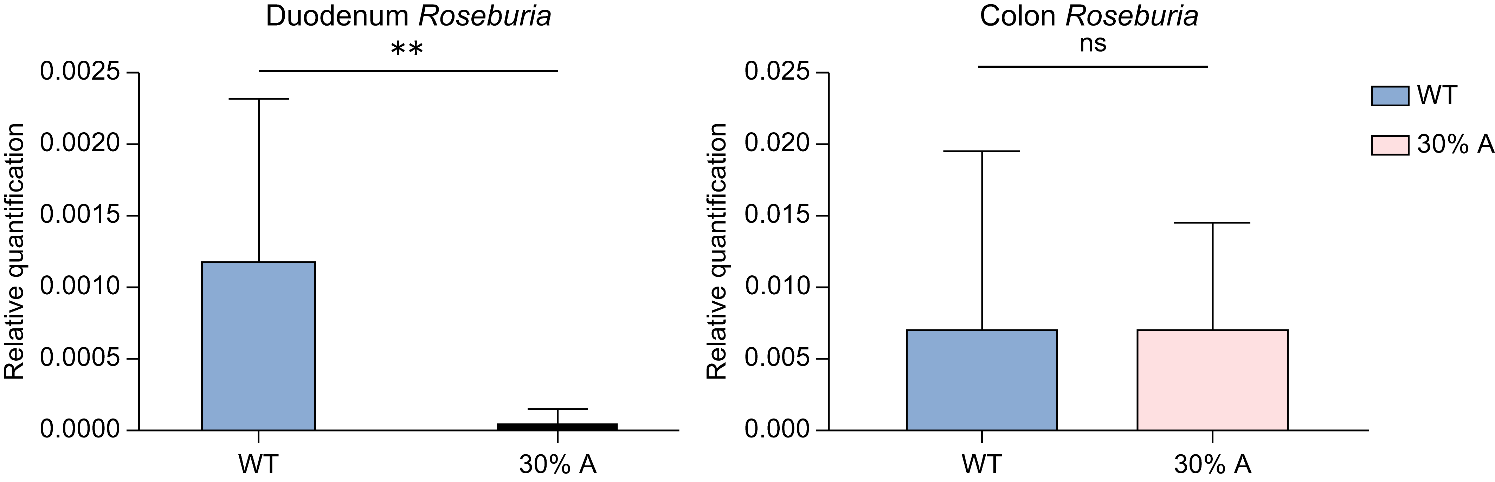
**

**Supplementary Figure 16.** Differences in *Roseburia* levels in the duodenum and colon of alcohol-consuming mice
